# Stool Mucosal Immune Protein–Cytokine Interconnections in Children with Autism Spectrum Disorder: An Age-Adjusted Partial Correlation Analysis

**DOI:** 10.3390/ijms27167097

**Published:** 2026-08-07

**Authors:** Joško Osredkar, Uroš Godnov, Maja Jekovec Vrhovšek, Damjan Osredkar, Gorazd Avguštin, Teja Fabjan, Kristina Kumer

**Affiliations:** 1 Institute of Clinical Chemistry and Biochemistry, University Medical Centre Ljubljana, Zaloška cesta 2, 1000 Ljubljana, Slovenia; 2 Faculty of Pharmacy, University of Ljubljana, Aškerčeva 7, 1000 Ljubljana, Slovenia; 3 Faculty of Mathematics, Natural Sciences and Information Technologies, University of Ljubljana, Glagoljaška ulica 8, 6000 Koper, Slovenia; 4 Centre for Autism, Unit of Child Psychiatry, University Children’s Hospital, University Medical Centre Ljubljana, Zaloška cesta 2, 1000 Ljubljana, Slovenia; 5 Department of Child, Adolescent and Developmental Neurology, Division of Pediatrics, University Medical Centre Ljubljana, Zaloška cesta 2, 1000 Ljubljana, Slovenia; 6 Faculty of Medicine, University of Ljubljana, Vrazov trg 2, 1000 Ljubljana, Slovenia; 7 Department of Microbiology, Biotechnical Faculty, University of Ljubljana, Groblje 3, 1230 Domžale, Slovenia

**Keywords:** autism spectrum disorder, stool proteomics, cytokines, IgA, calprotectin, alpha-1-antitrypsin, IL-1β, gut mucosal immunity, partial Spearman correlation, Luminex

## Abstract

Children with autism spectrum disorder (ASD) exhibit gut mucosal immune alterations, but the co-regulatory architecture linking stool immune proteins and cytokines within the same cohort remains unstudied. In 115 children (74 ASD, 41 controls; age 5–18 years), seven stool immune proteins (IgA subclasses, α_1_-antitrypsin and calprotectin subunits) were quantified by UHPLC-MS/MS and ten by Luminex—chemokines eotaxin/CCL11 and IL-8/CXCL8 plus eight cytokines—each normalised to total protein. Age-adjusted partial Spearman correlations were computed for all 70 protein–cytokine pairs per stratum, using Benjamini–Hochberg correction, bootstrap confidence intervals and Fisher r-to-z tests. No pair survived FDR correction in any stratum. IgA1 and IL-1β/TP correlated positively across all strata (full cohort ρ = 0.409, 95% CI 0.131–0.634, *n* = 62; controls 0.583; ASD 0.210), with no significant between-group difference (Fisher z = 1.70, *p* = 0.090). Multiple imputation attenuated this to ρ = 0.265 (95% CI 0.055–0.452). An inverse trend between IL-1β/TP and CARS score (ρ = −0.336, *n* = 41) did not survive correction (*p*-FDR = 0.576). This power-limited, hypothesis-generating study identifies an exploratory IgA1–IL-1β mucosal axis present across groups, with no confirmed between-group difference. Adequately powered multi-centre studies are required.

## 1. Introduction

Autism spectrum disorder (ASD) is a neurodevelopmental condition defined by persistent deficits in social communication and restricted, repetitive behavioural patterns. Its prevalence has risen substantially over recent decades; the most recent surveillance data from the United States estimate that 1 in 36 eight-year-old children meet the diagnostic criteria for ASD [1]. While the aetiology is multifactorial, converging evidence supports a role for systemic immune dysregulation as a contributor to ASD pathophysiology. Elevated pro-inflammatory cytokines, altered lymphocyte populations, and increased autoantibody titres have been described in the blood and cerebrospinal fluid of individuals with ASD [2], although the precise direction, magnitude, and consistency of these findings vary across studies.

The gut–immune axis has emerged as a particularly active area of ASD research over the past decade, motivated by the high prevalence of gastrointestinal symptoms in this population and by preclinical evidence that gut dysbiosis and altered mucosal immunity can influence neurodevelopment through microbiota–gut–brain pathways [3,4]. Epidemiological and microbiome studies have reported alterations in gut bacterial composition in ASD children, including reductions in Prevotella and Faecalibacterium species and increases in Clostridium and Desulfovibrio [5], although the causal direction of these associations remains unclear. These microbiota changes are likely to have downstream effects on mucosal immune homeostasis, including IgA class switching, cytokine production, and epithelial barrier function. However, direct measurement of mucosal immune proteins and cytokines in the same stool samples—allowing within-individual correlation analyses—has not previously been reported in an ASD cohort of this size.

A significant proportion of children with ASD present with gastrointestinal (GI) symptoms, with prevalence estimates ranging from 40% to 70% across published cohorts [3,6]. These symptoms include constipation, diarrhoea, bloating, and abdominal pain. Although the mechanistic underpinning of GI involvement in ASD remains incompletely characterised, converging lines of evidence point to alterations in intestinal permeability [7], gut microbiome composition, and local mucosal immune responses as candidate mechanisms. Critically, stool represents a non-invasive window onto the gut mucosal immune compartment and is therefore well suited for biomarker studies in paediatric populations.

Secretory immunoglobulin A (sIgA) is the principal mucosal antibody and a primary mediator of non-inflammatory intestinal defence. IgA exists in two subclasses: IgA1, which predominates in the systemic circulation and proximal intestine, and IgA2, which is enriched in the distal intestinal tract and is more resistant to bacterial proteolysis [8,9]. The regulation of IgA class switching in lamina propria B cells is orchestrated by a network of cytokines including IL-4, IL-10, TGF-β, IL-6, and IL-1β, the latter acting through NF-κB-dependent pathways [8,10]. Understanding how individual IgA subclass concentrations in stool relate to local cytokine levels within the same individual may illuminate whether cytokine-associated IgA regulation is potentially altered in children with ASD compared with neurotypical controls.

Calprotectin, a heterodimer of the calcium-binding proteins S100A8 and S100A9 (but also measurable as the S100A8 monomer, herein CAL-1), is released primarily from activated neutrophils and serves as a damage-associated molecular pattern (DAMP) [11]. It engages Toll-like receptor 4 (TLR4) and the receptor for advanced glycation end-products (RAGE), and its concentrations in stool are widely used as a surrogate marker of intestinal neutrophilic inflammation [12]. At the molecular level, calprotectin release is associated with local IL-1β signalling, as both share upstream activation of the NLRP3 inflammasome and NF-κB pathways. However, whether these associations between calprotectin and IL-1β are measurable in the stool of ASD children as compared with controls has not been formally examined.

Alpha-1-antitrypsin (A1AT), the predominant serine protease inhibitor in the human body, serves as both an acute-phase reactant and a marker of intestinal epithelial barrier integrity when measured in stool [7]. Elevated fecal A1AT concentrations are observed in protein-losing enteropathy and in conditions of heightened mucosal inflammation [13]. In the context of ASD, fecal A1AT measurements may reflect the low-grade intestinal inflammation and barrier permeability alterations that have been reported in this population [7,14].

The cytokine landscape in ASD is characterised by elevations in pro-inflammatory mediators including IL-1β, IL-6, and TNF, with variable findings for anti-inflammatory cytokines such as IL-10 and IL-4 [15,16]. Eotaxin (CCL11), a chemokine primarily associated with eosinophil recruitment, has been reported to be elevated in the plasma of individuals with ASD and has been proposed as a potential mediator of neuroinflammatory processes [17]. Mast cells are an important mucosal source of this chemokine and can modulate local vascular and lymphatic permeability, offering one route by which such mediators may act beyond the gut lumen [18]. The measurement of these cytokines in stool represents a more proximate reflection of gut mucosal immune activity than plasma measurements, yet stool cytokine data in ASD cohorts remain sparse. Beyond group-level biomarker discovery, improved identification of biological correlates of ASD—including in higher-functioning presentations that are often diagnosed later—may support earlier, more individualised clinical assessment when combined with standardised behavioural instruments [19].

Despite this growing body of evidence for gut mucosal immune involvement in ASD, no study to date has examined the correlational architecture between multiple stool immune proteins and multiple stool cytokines within the same cohort, using multivariate methods that account for the strong confounding effect of age on immune marker concentrations in a paediatric population spanning the range from early childhood to adolescence (5–18 years). The pairwise associations between proteins and cytokines—particularly after age adjustment—represent a fundamentally different question from simple group mean comparisons, and may reveal co-regulatory axes that are not apparent from univariate analyses.

The present study addresses three specific aims: (1) to characterise the age-adjusted partial Spearman correlations between seven stool immune proteins and ten stool cytokines in the full cohort and separately in ASD and control subgroups; (2) to identify any cytokine–protein axes with consistent signal across strata after FDR correction; and (3) to examine whether any protein or cytokine is associated with ASD severity as measured by the Childhood Autism Rating Scale (CARS). Formal Fisher r-to-z tests are used to evaluate whether correlation magnitudes differ significantly between ASD and control groups. The present report is part of a research programme including companion papers examining stool proteomics [20] and gut microbiome characteristics [21] in the same cohort, providing unique added value through within-subject cross-assay integration.

The broader context of this research programme is the need for multi-omic biomarker discovery in paediatric ASD that goes beyond single-analyte comparisons. Previous studies have typically compared mean concentrations of individual biomarkers between ASD and control groups using univariate tests, without accounting for the possibility that the co-regulatory architecture of the immune network may be altered even when individual analyte means are similar. The partial correlation approach taken here is specifically designed to capture these network-level relationships. A correlation-based analysis is not equivalent to, nor a substitute for, a group-comparison analysis: it addresses the question of whether two analytes co-vary within the same individual, conditional on age, not whether their mean levels differ between groups.

These three studies address distinct scientific questions that cannot be answered within a single manuscript: Osredkar et al. [20] examined protein abundance differences between ASD and control groups in a sibling-matched design; Finderle et al. [21] characterised cytokine concentration profiles across developmental age groups. Neither study examined the pairwise co-regulatory relationships between proteins and cytokines within the same individual—the correlational architecture that forms the specific focus of the present work. Multiplicity across the three papers is addressed at the stratum level: statistical corrections are applied within each paper independently, and the present study does not retest hypotheses from the companion works.

An important methodological consideration in the design of this study is the choice of the partial correlation approach over a more conventional network analysis (e.g., graphical LASSO, partial correlation networks). The choice of pairwise partial correlations controlled for age—rather than network-level regularisation—was made deliberately for interpretability and statistical transparency: each coefficient has a direct, well-understood biological interpretation (the conditional association between protein X and cytokine Y given age), and the FDR correction is straightforward to apply and interpret. Network regularisation methods, while potentially more powerful for identifying hub nodes in high-dimensional data, require sample sizes substantially larger than those available in this study and impose assumptions about network structure that are not testable in a dataset of this size.

## 2. Results

### 2.1. Cohort Characteristics

The study enrolled 115 children (ASD *n*  =  74, controls *n*  =  41) aged 5–18 years. Participant flow is summarised in Supplementary Figure S1 (STROBE item 13). Demographic and clinical characteristics are shown in Table 1. The ASD group had a median age of 9.8 years (IQR 6.7–12.8 years), with 59 males and 15 females (79.7% male). The control group had a median age of 10.8 years (IQR 7.8–12.6 years), with 21 males and 20 females (51.2% male). Age did not differ significantly between groups (Mann–Whitney U, *p*  =  0.317). The sex distribution differed significantly (χ^2^  =  8.82, *p*  =  0.003), consistent with the known male predominance in ASD, and was addressed in sensitivity analyses (Section 2.5). The Childhood Autism Rating Scale (CARS) was available for all 74 children in the ASD group, with a median score of 35.2 (IQR 29.5–40.0); 22 children scored below 30 and were retained in the ASD group on the basis of confirmed DSM-5 diagnosis, and 52 scored 30 or above.

### 2.2. Distributional Properties and Normality Testing

Shapiro–Wilk normality testing was performed for all 17 analytes (7 proteins and 10 cytokines, each normalised to total protein). Sixteen of 17 markers showed significant deviation from normality (*p*  <  0.0002), confirming that parametric analyses would be inappropriate for this dataset and providing the primary justification for the non-parametric partial correlation approach. One marker—Eotaxin/TP—was borderline (W  =  0.973, *p*  =  0.055) and considered approximately normally distributed; however, the non-parametric approach was retained for consistency across all comparisons. Substantial pairwise missing data were observed, particularly for IL-4 (~38% missing) and IL-10 (~44% missing), and for Vitros total protein measurements (*n*  =  25 samples missing, 21.7%), which were required for cytokine normalisation. All correlations used pairwise deletion. Descriptive statistics for all markers, stratified by group, are presented in Table 2.

**Table 2:** Descriptive statistics for stool immune proteins and cytokines (median [IQR]) by group.

Marker (Unit)	ASD Median [IQR]	*n*	Control Median [IQR]	*n*	*p*
Stool proteomics (RECETOX; ng/mg total stool protein)					
IgA1 (ng/mg)	670.9 [316.0–1237.2]	57	506.5 [266.3–970.8]	36	0.342
IgA2 (ng/mg)	1086.1 [539.0–2467.6]	68	1191.0 [684.1–2248.4]	40	0.719
IgA1+2 (ng/mg)	1705.4 [759.5–3963.1]	72	1858.6 [1153.4–3678.6]	40	0.351
A1AT-1 (ng/mg)	137.2 [94.6–191.7]	71	123.3 [86.7–197.7]	40	0.729
CAL-1 (ng/mg)	30.5 [16.9–72.2]	70	25.6 [13.9–39.3]	39	0.115
CAL-2 (ng/mg)	50.6 [29.9–111.3]	62	41.5 [22.2–54.5]	33	0.218
CAL1+2 (ng/mg)	86.9 [51.0–191.3]	62	70.0 [37.9–95.4]	33	0.178
Stool cytokines (Luminex; pg/mg total stool protein)					
Eotaxin/TP (pg/mg)	0.116 [0.062–0.162]	52	0.128 [0.077–0.160]	37	0.603
IFN-γ/TP (pg/mg)	1.736 [0.815–2.817]	42	1.366 [0.685–2.108]	32	0.365
IL-1α/TP (pg/mg)	58.5 [26.8–102.4]	53	53.1 [21.5–87.1]	37	0.555
IL-1β/TP (pg/mg)	0.064 [0.021–0.128]	41	0.061 [0.039–0.140]	33	0.514
IL-4/TP (pg/mg)	0.108 [0.054–0.158]	36	0.103 [0.060–0.182]	19	0.993
IL-6/TP (pg/mg)	0.236 [0.152–0.352]	44	0.192 [0.102–0.318]	33	0.140
IL-8/TP (pg/mg)	0.165 [0.081–0.290]	42	0.158 [0.081–0.351]	32	0.857
IL-10/TP (pg/mg)	0.380 [0.162–0.867]	32	0.341 [0.105–0.884]	18	0.664
IL-17/TP (pg/mg)	0.738 [0.422–1.443]	52	0.623 [0.426–1.092]	36	0.458
TNF/TP (pg/mg)	2.920 [1.214–7.310]	40	1.723 [0.686–3.841]	31	0.249

(1) All values are medians [interquartile range]; n is the number of samples with a valid measurement for that marker in that group and varies by marker because of pairwise missingness. (2) Stool proteins are normalised to BCA-measured total protein (ng/mg) and cytokines to Vitros-measured total protein (pg/mg TP); the two denominators originate in different laboratories and their concordance is reported in Section 4.4. (3) *p*-values are from Mann–Whitney U tests comparing ASD with controls for that marker, uncorrected for multiple comparisons; no marker reached significance. (4) These are descriptive group comparisons and answer a different question from the partial correlations in Table 3, Table 4 and Table 5, which concern within-individual co-variation rather than group means. (5) Complete distributional statistics and Shapiro–Wilk results are given in Supplementary Table S5. TP: total protein.

**Table 3:** Top protein–cytokine associations in the full cohort (age-adjusted partial Spearman, 70-pair FDR family). Sig.: † *p*-FDR < 0.10 (trend); no pair reached *p*-FDR < 0.05.

Protein	Cytokine	ρ	95% CI	*n*	*p*-Raw	*p*-FDR	Sig.
IgA1	IL-1β/TP	0.409	[0.131–0.634]	62	0.001	0.075	†
IgA1+2	IL-1β/TP	0.309	[0.066–0.511]	72	0.009	0.308	ns
CAL-1	IL-1β/TP	0.295	[0.042–0.514]	71	0.013	0.309	ns
A1AT-1	IL-1β/TP	0.262	[0.026–0.469]	73	0.026	0.464	ns
A1AT-1	Eotaxin/TP	0.228	[0.036–0.409]	88	0.034	0.472	ns

**Figure 1:**
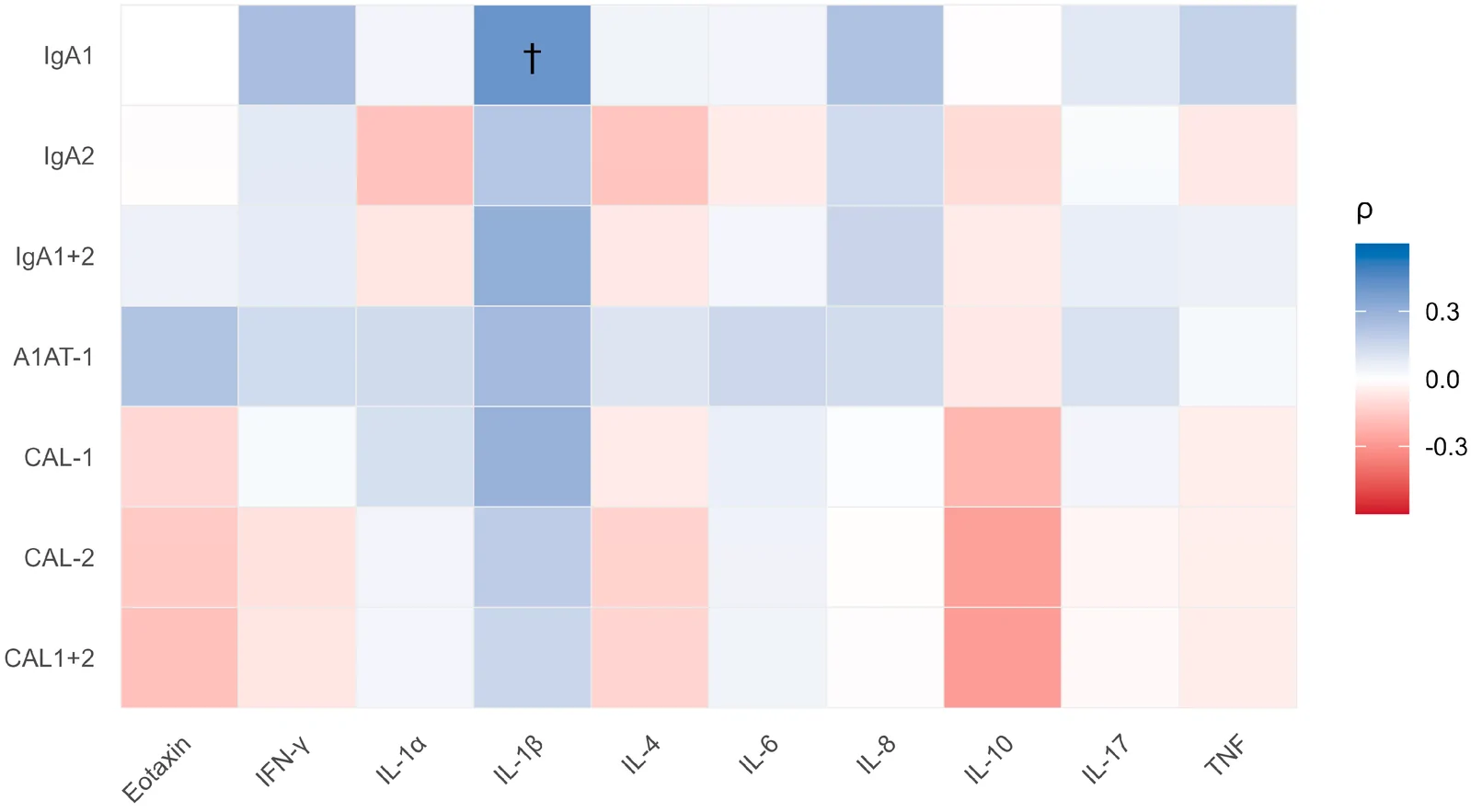
Age-adjusted partial Spearman correlation coefficients (ρ) between seven stool immune proteins (rows) and ten stool cytokines and chemokines (columns) in the full cohort. Colour indicates the magnitude and direction of ρ (blue positive, red negative). Because pairwise deletion was used, the effective sample size differs between cells and ranges from *n* = 36 to *n* = 89. No cell reached statistical significance after Benjamini–Hochberg correction across the 70-pair family; the strongest association (IgA1–IL-1β/TP, ρ = 0.409, *p*-FDR = 0.075) is marked † (*p*-FDR < 0.10, trend).

(1) ρ is the age-adjusted partial Spearman correlation coefficient, computed by the Conover–Iman rank transform (Section 4.7). (2) CI is the bootstrap 95% confidence interval, percentile method, 2000 resamples. (3) n is the pairwise effective sample size, which differs across pairs because pairwise deletion was used; the consequences are discussed in Section 3.7, item 3. (4) *p*-raw is the two-tailed *p*-value before correction. (5) *p*-FDR is the Benjamini–Hochberg adjusted *p*-value computed across all 70 protein–cytokine pairs of the full-cohort stratum (7 proteins × 10 cytokines). (6) The Sig. column shows † for *p*-FDR < 0.10 (trend) and ns otherwise; no pair in this stratum reached *p*-FDR < 0.05. (7) Only the five pairs with the smallest *p*-raw are listed; the complete 70-pair matrix is shown in Figure 1.

**Table 4:** Group-specific protein–cytokine associations and between-group Fisher r-to-z comparison (IgA1–IL-1β/TP).

Protein	Cytokine	ρ	95% CI	*n*	*p*-Raw	*p*-FDR	Sig.	Group
IgA1	IL-1β/TP	0.583	[0.169–0.822]	30	<0.001	0.063	†	Control
IgA1+2	IL-1β/TP	0.424	[0.086–0.672]	32	0.018	0.457	ns	Control
IgA2	IL-1α/TP	−0.393	—	36	0.020	0.457	ns	Control
A1AT-1	Eotaxin/TP	0.288	[0.027–0.525]	51	0.042	0.961	ns	ASD
IgA1	IL-1β/TP	0.210	[−0.190–0.577]	32	0.256	0.961	ns	ASD

(1) Correlations are age-adjusted partial Spearman coefficients computed separately within each diagnostic group, so the ASD and control strata form separate correction families. (2) These strata are not independent of the full-cohort family in Table 3, since the full cohort is the union of the two subgroups; a pair may therefore appear in more than one family, and the strata should not be read as independent replications. (3) The Sig. column shows ns for not significant after FDR correction, † for *p*-FDR < 0.10. (4) The between-group comparison of the IgA1–IL-1β/TP coefficient is a Fisher r-to-z test: z = 1.70, *p* = 0.090. The difference is a trend that does not reach significance, so the numerically higher coefficient in controls must not be read as evidence of altered coupling in ASD; a difference in significance between two groups is not itself a significant difference [22]. (5) The test is a single exploratory comparison, not part of any correction family. ρ: partial Spearman rho; CI: 95% bootstrap CI. The *p*-FDR values in this table have been recomputed across the 70-pair family declared in Section 4.7, matching Table 3; the values in the submitted version had been adjusted within a 10-test family, which is why the control-group IgA1–IL-1β/TP association previously appeared to survive correction. On the declared family it does not, and no pair is significant after FDR correction in any stratum.

**Table 5:** Age-adjusted partial Spearman correlations between stool markers and CARS total score (ASD subgroup, *n* per marker varies).

Marker	ρ	*n*	*p*-Raw	*p*-FDR	Interpretation
IL-1β/TP	−0.336	41	0.034	0.576	Nominal only
TNF/TP	−0.204	40	0.213	0.995	—
IgA1	−0.156	57	0.251	0.995	—
IL-1α/TP	0.132	53	0.350	0.995	—
IL-10/TP	−0.123	32	0.511	0.995	—
CAL-1	0.107	70	0.382	0.995	—
IL-4/TP	−0.091	36	0.604	0.995	—
Eotaxin/TP	0.077	52	0.593	0.995	—
IgA1+2	−0.076	72	0.529	0.995	—
A1AT-1	−0.064	71	0.600	0.995	—
IFN-γ/TP	−0.056	42	0.726	0.995	—
IgA2	−0.050	68	0.687	0.995	—
CAL-2	−0.035	62	0.787	0.995	—
IL-8/TP	−0.028	42	0.860	0.995	—
IL-17/TP	−0.017	52	0.904	0.995	—
IL-6/TP	0.006	44	0.968	0.995	—
CAL1+2	−0.001	62	0.995	0.995	—

(1) ρ is the age-adjusted partial Spearman correlation between the stool marker and CARS total score within the ASD subgroup. (2) CARS was available for all 74 ASD children, so the maximum possible n is 74; n varies by marker because of missing analyte data, chiefly the unavailability of Vitros total protein in 21.7% of samples (Section 4.4). (3) *p*-raw is the uncorrected two-tailed *p*-value; *p*-FDR is the Benjamini–Hochberg adjusted value across the family of 17 marker tests—a different and smaller family than the 70-pair family used in Table 3 and Table 4. (4) “Nominal only” marks an association reaching *p*-raw < 0.05 that does not survive FDR correction; such rows carry no evidentiary weight and are reported for completeness. (5) Rows are ordered by descending absolute ρ.

### 2.3. Protein–Cytokine Associations: Full Cohort

Age-adjusted partial Spearman correlations were computed for all 70 protein–cytokine pairs (7 proteins × 10 cytokines) in the full cohort (*n* = 36–89 per pair, depending on pairwise availability). Benjamini–Hochberg FDR correction was applied within each analytical stratum independently, using 70 pairs as the correction family per stratum. After Benjamini–Hochberg FDR correction across all 70 protein–cytokine pairs in the full-cohort stratum, no pair reached statistical significance at α = 0.05. The strongest association, IgA1–IL-1β/TP (ρ = 0.409, *p*-raw = 0.001), yielded a *p*-FDR of 0.075, representing a statistical trend. The top associations are summarised in Table 3 and Supplementary Figure S3. Bootstrap 95% confidence intervals (2000 resamples, percentile method) were computed for the pairs showing the most consistent positive associations, all involving IL-1β/TP as the cytokine partner.

IL-1β/TP emerged as the cytokine most consistently associated with immune proteins in the full cohort. No other cytokine yielded associations of comparable magnitude or consistency. The protein–cytokine pairs listed in Table 3 all displayed positive associations, suggesting a pattern of co-variation between IgA markers and neutrophil-derived proteins on one hand and IL-1β on the other All findings must be treated as exploratory trends, as no pair survived Benjamini–Hochberg FDR correction across all 70 protein–cytokine pairs in the full-cohort stratum.

Among the ten cytokines assessed, eight showed fewer than two nominally significant associations (*p*-raw  <  0.05) with any protein in the full cohort: IFN-γ/TP, IL-1α/TP, IL-4/TP, IL-6/TP, IL-8/TP, IL-10/TP, IL-17/TP, and TNF/TP. The pattern of consistently weak associations with these cytokines is consistent with the hypothesis that the association between mucosal immune proteins and local cytokine concentrations in stool is largely specific to the IL-1β axis in this age- and condition-diverse cohort, at least at the effect sizes accessible with the current sample size. Eotaxin/TP showed a modest association with A1AT-1 (ρ  =  0.228, *p*-FDR  =  0.472), which is consistent with eosinophilic mucosal involvement and barrier dysfunction but does not achieve FDR significance.

The heat map of all 70 full-cohort partial correlations (Figure 1) shows a gradient of positive associations concentrated in the IL-1β column and the IgA1, IgA1+2, CAL-1, and A1AT-1 rows. The remaining matrix entries cluster near zero, with only three negative correlations exceeding |ρ|  >  0.10 (IgA2–IL-1α/TP in controls: ρ  =  −0.393), none of which survived FDR correction. This distributional pattern—positive IL-1β associations concentrated in specific protein rows while other matrix entries approach zero—argues against a global inflation of correlations due to shared measurement error, and supports a degree of analyte-pair specificity.

### 2.4. Subgroup Analysis: ASD and Control Groups

Age-adjusted partial Spearman correlations were computed separately for the ASD (*n*  =  32–74 per pair) and control *(n*  =  30–41 per pair) subgroups across the same pair list. No pair reached FDR-corrected significance in either stratum Table 4; Supplementary Figure S2). In the ASD subgroup, the IgA1–IL-1β/TP partial correlation was ρ  =  0.210 (95% CI −0.190–0.577, *n*  =  32, *p*-FDR  =  0.961), compared with ρ  =  0.583 (95% CI 0.169–0.822, *n*  =  30, *p*-FDR  =  0.063) in controls. The between-group difference approached but did not reach significance (Fisher r-to-z: z  =  1.70, *p*  =  0.090), indicating that the apparent divergence in FDR outcomes partly reflects differential statistical power (*n*  =  32 vs. *n*  =  30 effective pairs with IL-1β).

### 2.5. Sensitivity Analysis: Sex as Additional Covariate

Given the significant sex imbalance between groups (χ^2^  =  8.82, *p*  =  0.003), an exploratory sensitivity analysis was performed by adding sex as a second covariate (controlling for both age and sex) using the rank-partial-correlation method extended to two covariates. Results were consistent with the age-only analysis. Full sex-adjusted statistics for the IgA1–IL-1β/TP pair were: full cohort, ρ = 0.402 (95% CI 0.125–0.644, *n* = 62, *p*-raw = 0.001, *p*-FDR = 0.102); controls, ρ = 0.579 (95% CI 0.164–0.838, *n* = 30, *p*-raw = 0.001, *p*-FDR = 0.088); ASD, ρ = 0.205 (95% CI −0.177–0.585, *n* = 32, *p*-raw = 0.278, *p*-FDR = 0.950). These estimates are essentially unchanged from the age-only model, indicating that sex imbalance does not account for the observed associations. Formal sex-stratified analysis (separate correlation models within males and within females) was not performed, as the resulting subgroup sizes (e.g., *n* = 15 ASD females) are too small to yield stable partial-correlation estimates; this is acknowledged as a limitation (Section 3.7, item 5). In the full cohort, ASD subgroup, and control subgroup, zero protein–cytokine pairs achieved FDR-corrected significance. The direction and approximate magnitude of associations were qualitatively consistent with the age-only model.

### 2.6. Sensitivity Analysis: Multiple Imputation for Missing Cytokine Data

Given the substantial missingness for IL-4 (~38%), IL-10 (~44%) and Vitros total protein (unavailable in 21.7% of samples and required for cytokine normalisation), a sensitivity analysis using multiple imputation by chained equations (MICE; R package mice, m = 30 imputations, predictive mean matching, 20 iterations) was performed for the two associations central to this manuscript: IgA1–IL-1β/TP (full cohort) and CARS–IL-1β/TP (ASD subgroup). Imputation models included age, sex, diagnostic group and all 17 markers as predictors. Partial Spearman coefficients were pooled across imputed datasets on the Fisher z scale and back-transformed to the correlation scale [23]. Pooled estimates were: IgA1–IL-1β/TP, ρ = 0.265 (95% CI 0.055–0.452, *n* = 115, *m* = 30), against ρ = 0.409 (*n* = 62) under pairwise deletion; and CARS–IL-1β/TP, ρ = −0.202 (95% CI −0.451–0.076, *n* = 74, *m* = 30), against ρ = −0.336 (*n* = 41) under pairwise deletion. Both imputed estimates retain the direction of the pairwise-deletion results but are attenuated by roughly one third in magnitude, and the confidence interval for the CARS–IL-1β/TP association now includes zero. The imputation therefore does not corroborate the pairwise-deletion estimates so much as qualify them: the most plausible reading is that complete-case analysis of these particular associations is upward-biased, consistent with a departure from the missing-completely-at-random assumption. Because values below the assay detection limit are systematically associated with truly low concentrations, a missing-not-at-random mechanism cannot be excluded and multiple imputation does not fully correct for it. This reinforces rather than weakens the exploratory framing applied throughout.

### 2.7. CARS Severity Analysis

CARS scores were available for all 74 ASD children; age-adjusted partial Spearman correlations were computed between each of the 17 stool markers and CARS total score (*n*  =  32–74 per marker, depending on pairwise availability of the analyte). No marker achieved FDR-corrected significance after Benjamini–Hochberg correction across 17 marker tests. The numerically most notable trend was an inverse partial correlation between IL-1β/TP and CARS score (ρ  =  −0.336, 95% CI −0.606–0.000, *n*  =  41, *p*-raw  =  0.034; *p*-FDR  =  0.576) (Supplementary Figure S4). The effective sample size for this analysis was limited to *n*  =  41 after pairwise deletion for IL-1β/TP missingness, despite CARS being available for all 74 ASD children; the discrepancy reflects the exclusion of IL-1β results reported below the assay detection limit (19.1% of the analysed cohort; Supplementary Table S3), compounded by the unavailability of Vitros total protein, required for normalisation, in a further subset of samples. A weaker inverse trend was observed for TNF/TP (ρ  =  −0.204, *n*  =  40, *p*-raw  =  0.213; *p*-FDR  =  0.995). All other markers yielded *p*-FDR  ≥  0.90 (Table 5). These findings are treated as hypothesis-generating only and do not support conclusions regarding the relationship between fecal cytokine concentrations and ASD severity.

The CARS total scores in the present cohort ranged from 15.0 to 60.0 (median 35.2, IQR 29.5–40.0). The distribution was approximately unimodal with a mode in the mild-to-moderate range (30–40). The sub-classification of 22 children scoring below 30 as non-autistic by the standard CARS cut-point does not imply that these children lack ASD: all were diagnosed by a certified child psychiatrist using DSM-5 criteria, which may not align with the older CARS scoring conventions. These 22 children were retained in the ASD group for all analyses, and their exclusion in a sensitivity analysis did not materially change the IL-1β–CARS partial correlation (ρ  =  −0.291, *n*  =  32, *p*  =  0.113).

## 3. Discussion

### 3.1. Principal Findings

We state at the outset that the central result of this study is a null finding: no stool protein–cytokine pair, in any analytic stratum, survived correction for multiple comparisons, and none of the associations discussed below should be interpreted as confirmed biological signals. The primary finding of this study is that, after age adjustment, no stool immune protein–cytokine pair achieved statistical significance following Benjamini–Hochberg FDR correction across 70 protein–cytokine pairs per stratum (7 proteins × 10 cytokines), in the full cohort, ASD subgroup, control subgroup, or CARS severity analysis. This is an honest null result, and its interpretation requires careful consideration of statistical power, missingness, and the exploratory nature of the analysis. The null finding across the full 70-pair FDR family is important and should not be misread as equivalent to a finding of “no association”: the study was substantially underpowered for many pairs, and the absence of significance is expected given the sample sizes and the number of tests.

Three analytic strata (full cohort, ASD, controls) and one exploratory stratum (CARS severity) were examined. The hierarchy of evidence is: (1) full cohort associations represent the most powered analyses and are the primary focus; (2) subgroup associations in ASD and controls are secondary and exploratory; (3) between-group Fisher r-to-z tests are exploratory tests for subgroup differences but should be interpreted as single tests rather than a family; (4) CARS associations are entirely exploratory. This analytic hierarchy was defined by the study team prior to inspection of the correlation results but was not deposited in a public trial registry or repository (e.g., ClinicalTrials.gov, OSF) prior to analysis. In the absence of timestamped, publicly available registration, this hierarchy cannot be considered a confirmatory pre-registered analysis plan; it should be regarded as an internally defined analytic framework, and all associated findings—including those labelled ‘primary’—are exploratory in nature.

Within this context of no FDR-corrected significance, a consistent positive partial correlation between IgA1 and IL-1β/TP—observed across all three analytical strata (full cohort, ASD, controls)—represents the single most reproducible exploratory signal in the dataset. The confidence intervals across strata overlap substantially, and formal Fisher r-to-z testing confirms the absence of a statistically significant between-group difference in this association (z  =  1.70, *p*  =  0.090). The numerically lower correlation in the ASD subgroup (ρ  =  0.210 versus ρ  =  0.583 in controls) should not be interpreted as evidence of a genuine group-specific modulation of this association; it is plausibly explained by reduced sample size (*n*  =  32 versus *n*  =  30 for this pair) and consequently wider confidence intervals that cross zero.

### 3.2. The IgA1–IL-1β Exploratory Mucosal Axis

The biological plausibility of a positive association between IgA1 and IL-1β in the intestinal compartment is well established. IL-1β is a potent activator of NF-κB signalling in lamina propria B cells, and NF-κB activation is required for the Ig heavy-chain class switch recombination that generates IgA-secreting plasma cells [8,10,24]. In the intestinal mucosa this pathway is engaged by several upstream sensors, including NOD-like receptors signalling through RIP2 [25], so local NF-κB tone reflects more than IL-1 receptor engagement alone. A local environment with elevated IL-1β may therefore be permissive for, or associated with, higher IgA1 production—reflecting an activated but not yet suppressed mucosal immune state. The observation that this association is present and positive in both ASD children and neurotypical controls (with overlapping confidence intervals) is consistent with a general feature of mucosal immune biology rather than an ASD-specific phenomenon.

This association should be regarded as an exploratory hypothesis—the IgA1–IL-1β co-variation as a candidate correlational hub in this dataset—rather than a confirmed biological finding. Its validation requires a prospectively designed, adequately powered study with pre-specified primary endpoints. The present data provide the effect size estimates needed to power such a study: using the full-cohort ρ  =  0.409 with *n*  =  62 as the basis, a future study targeting 80% power at α  =  0.05 for a single primary pair would require approximately 50–55 complete pairs, achievable in a multi-centre design.

The observation that the IgA1–IL-1β association is present in both the ASD and control groups with positive directional consistency (ρ  =  0.210 and ρ  =  0.583, respectively) has implications for biomarker discovery strategy. If this association represents a general mucosal immune regulatory relationship—present in both neurotypical and ASD children—then it cannot serve as a discriminating biomarker for ASD diagnosis. However, the numerically weaker association in ASD (which may reflect the smaller effective sample size or a genuine partial attenuation) could become informative in a larger study. A confirmed reduction in the IgA1–IL-1β coupling strength in ASD relative to controls would suggest potential alterations in mucosal immune co-regulation that are not apparent from mean comparisons alone.

### 3.3. Calprotectin and A1AT Associations

Consistent positive associations with IL-1β/TP were also observed for CAL-1 (ρ  =  0.295, 95% CI [0.042–0.514]) and A1AT-1 (ρ  =  0.262, 95% CI [0.026–0.469]) in the full cohort, both failing FDR correction. The co-variation of calprotectin (S100A8 monomer) with IL-1β is biologically coherent: both are products of activated neutrophils and macrophages, and both are associated with inflammasome activation and NF-κB-mediated signalling [11,12].

Only CAL-1 (S100A8 monomer), and not the S100A9 monomer (CAL-2) or the S100A8/S100A9 heterodimer (CAL1+2), showed a nominal positive association with IL-1β/TP; the mechanistic implications of this subunit specificity are discussed as a future research priority in Section 3.9.

Similarly, A1AT, as an acute-phase protein whose intestinal concentrations reflect barrier integrity, is expected to track with local inflammatory mediators such as IL-1β [7]. However, IL-1β activates NF-κB and MAPK pathways—rather than STAT3, which is the primary signalling pathway for IL-6/gp130—and this may upregulate A1AT expression locally in intestinal epithelium; the contribution of hepatic acute-phase synthesis to fecal A1AT levels is likely minor compared with local barrier leakage. These associations were not significant after FDR correction and should be treated as preliminary observations.

### 3.4. Absence of FDR-Significant Findings: Interpretation and Power

The absence of FDR-significant protein–cytokine pairs across all strata is not unexpected given the statistical context of the study. The subgroup analyses involved 30–74 participants per stratum, with pairwise missingness further reducing effective sample sizes for several analyte pairs (notably those involving IL-4 and IL-10, with missingness of 38% and 44%, respectively). With 70 protein–cytokine pairs per FDR family to correct across within each stratum, and subgroup sample sizes of 30–40 for the control group, the study was underpowered to detect anything other than very large correlation coefficients at FDR-corrected significance thresholds. The absence of significance therefore does not imply the absence of biologically meaningful associations [22].

This framing is consistent with the STROBE guidelines for reporting observational studies [26]: the primary value of the present analysis is in providing precisely estimated (via bootstrap) effect sizes and formally tested between-group comparisons, rather than in generating definitively confirmed biomarker findings. The use of Benjamini–Hochberg correction ensures that the null findings are not an artefact of overly conservative correction. Power calculations post hoc confirm the study had approximately 50% power to detect a true ρ  =  0.30 at FDR α  =  0.05 in the full cohort, and substantially less power in the ASD and control subgroups. The effect size landscape observed here—with most partial correlations between 0.20 and 0.41—is consistent with the range expected for cytokine–protein associations in gut biology.

### 3.5. CARS Severity Trend

The nominal inverse partial correlation between IL-1β/TP and CARS total score (ρ  =  −0.336, *n*  =  41, *p*-raw  =  0.034; *p*-FDR  =  0.576) is an exploratory finding that should be interpreted with caution. An inverse association could suggest that children with higher fecal IL-1β concentrations have lower (less severe) CARS scores. Potential explanations include a compensatory upregulation of anti-inflammatory IL-1 receptor antagonist (IL-1Ra) in more severely affected children, or systematic differences in gut permeability across the CARS spectrum.

However, since neither IL-1Ra nor IL-10 was elevated in our dataset in association with CARS, the compensatory hypothesis remains speculative. Alternative explanations include chance (this nominal association does not survive FDR correction, *p*-FDR  =  0.576), dietary factors or restricted eating patterns common in severe ASD that may suppress inflammatory markers, gastrointestinal status varying with symptom severity [27], reverse causation, or the possibility that the most severely affected children represent a neuroinflammatory-predominant phenotype with relatively suppressed peripheral gut inflammation. This trend is hypothesis-generating and does not support clinical conclusions.

### 3.6. Strengths

Several methodological strengths distinguish this study from prior work in the field. First, this is, to the best of our knowledge, the first study to measure both stool immune proteins by UHPLC-MS/MS proteomics and stool cytokines by multiplex Luminex assay in the same children at the same time point, enabling within-subject cross-assay correlational analysis that is free from the batch and temporal sources of variation that would affect cross-cohort comparisons. The paired design ensures that correlations reflect genuine within-individual co-regulatory relationships rather than between-individual confounding.

Second, the use of age as a continuous covariate in partial Spearman correlations is appropriate for a paediatric cohort spanning a 13-year age range (5–18 years), and avoids the distortions introduced by age-stratification with small subgroups. The Conover-Iman rank-transform method [28] is a validated and computationally stable approach to non-parametric partial correlation that has been validated against the ppcor reference implementation. Third, bootstrap 95% confidence intervals (2000 resamples, percentile method) provide directly interpretable measures of uncertainty around each correlation estimate, enabling effect size planning for future studies.

Fourth, the Fisher r-to-z test provides a formal statistical framework for the between-group comparison of correlation magnitudes, resolving the question of whether the IgA1–IL-1β association differs between ASD and control children with a single, formally defined test statistic rather than informal visual comparison of confidence intervals. Fifth, transparent null reporting—with FDR correction applied across all 70 protein–cytokine pairs per stratum and results presented without selective emphasis on nominally significant trends—is consistent with current best practices in biomarker research and with the STROBE statement [26].

Sixth, the inclusion of CARS severity scores for all 74 ASD children enables an exploratory analysis of biomarker–severity relationships. The availability of CARS for the entire ASD sample (rather than a subset) maximises statistical power for the severity analysis and reduces the risk of selection bias in the CARS subsample. Seventh, the use of UHPLC-MS/MS proteomics with stable isotope-labelled internal standards provides high analytical accuracy and selectivity for the quantification of IgA subclasses and other immune proteins, which would be difficult to distinguish by conventional immunoassay.

### 3.7. Limitations

The following limitations should be considered in interpreting these results:

1. Cross-sectional design. No causal inference can be drawn from correlation analyses. The observed associations between proteins and cytokines may reflect shared upstream regulatory mechanisms, parallel responses to the same environmental stimuli, or methodological co-variation.
2. Single centre, tertiary referral setting. Children attending a specialist centre may not be representative of the broader ASD population.
3. High cytokine missingness and use of pairwise deletion. IL-4 (~38% missing), IL-10 (~44% missing) and total protein for cytokine normalisation (~21.7% missing) showed substantial missingness, and IL-1β fell below the assay LOD in 19.1% of samples. All correlation analyses used pairwise deletion, which assumes data are missing completely at random (MCAR)—an assumption unlikely to hold fully, since missingness in stool cytokines may relate to stool consistency, processing delay, or assay performance near the detection limit. Pairwise deletion also produces varying effective sample sizes across pairs (*n* = 36–89), which complicates cross-pair and cross-group comparison and affects the precision of Fisher r-to-z tests. A multiple-imputation sensitivity analysis (Section 2.6) for the two key associations returned estimates in the same direction but roughly one third smaller, with the CARS–IL-1β/TP interval crossing zero. Complete-case analysis of these associations is therefore likely to be upward-biased, and the exploratory estimates reported here should be read as upper bounds rather than as point estimates of a stable effect. A fully MAR- or MNAR-robust analysis of all 70 pairs was beyond the scope of this study [23].
4. Cytokine assay validity in the stool matrix. The validity of Luminex-measured cytokines in stool homogenates has not been formally established for all analytes in this matrix. The percentage of samples above the assay LOD, and the smaller percentage falling within the fully quantifiable (above-LOQ) range, are reported for every analyte and diagnostic group in Supplementary Table S3. Across the analysed cohort the proportion above the LOD ranged from 56.5% (IL-10) and 59.1% (IL-4) to above 98% for IL-1α, Eotaxin/CCL11 and IL-17, but the proportion within the fully quantifiable range was markedly lower for several analytes, notably IL-1β (80.9% above LOD but only 20.0% above LOQ) and IL-8/CXCL8 (82.6% and 18.3%). For several analytes a substantial share of detectable values fell below the LOQ and were therefore extrapolated below the validated standard curve. IL-1β is known to be proteolytically labile, and the proteolytic stool environment may cause variable degradation independent of true biological concentration. All samples were subjected to a maximum of two freeze–thaw cycles (Section 4.4), but formal spike-recovery experiments and matrix-specific freeze–thaw stability data for IL-1β and the other analytes in stool homogenate were not generated as part of this study and are not available in the published literature for this specific matrix. This represents an unresolved analytical limitation: we cannot formally exclude the possibility that some of the observed correlations, particularly those involving IL-1β, are partly influenced by differential analyte recovery or degradation rather than purely reflecting underlying biology.
5. Sex imbalance. Sex distribution differed significantly between groups (79.7% male in ASD vs. 51.2% in controls; χ^2^  =  8.82, *p*  =  0.003). Although sex was included as a covariate in a sensitivity analysis and did not materially change results, sex-stratified analyses were underpowered given current sample sizes. Future studies should recruit sex-balanced cohorts or stratify analyses by sex.
6. Dietary confounders. No systematic data on diet, dietary supplements (including omega-3 fatty acids, zinc, and vitamins known to modulate S100 proteins and IL-1β), or restrictive eating behaviours were collected. These are likely differentially distributed between ASD and control groups and represent uncontrolled confounders.
7. Underpowered subgroup analyses. The control subgroup (*n*  =  30–41 per pair after missingness) was particularly underpowered for the detection of moderate correlations (ρ  ≈  0.3–0.4) at FDR-corrected thresholds.
8. Exploratory between-group test. No formal test of the difference between correlation coefficients in ASD versus controls was pre-specified as a primary endpoint; the Fisher r-to-z analysis (z  =  1.70, *p*  =  0.090) was exploratory.
9. No independent replication cohort. All findings should be considered unconfirmed until independently replicated in a separate cohort using a comparable analytic framework.

### 3.8. Considerations on Sex, Age, and Cohort Heterogeneity

The present cohort spans a wide age range (5–18 years) and includes children from both early childhood and adolescence. The choice of a continuous age covariate, rather than age-group stratification, preserves statistical power while controlling for the well-documented age-related changes in gut mucosal immune marker concentrations that occur during development. Immunoglobulin A concentrations in stool increase with age from infancy through adolescence, reaching near-adult levels by approximately 10–12 years; IL-1β and other pro-inflammatory cytokines show more variable age trajectories. Controlling for age as a continuous variable is therefore essential to avoid spurious correlations driven by shared age dependence of both analytes. The partial correlations reported here are partial of age: they represent the residual association between protein and cytokine after both have been adjusted for age.

### 3.9. Future Directions

The findings of this study motivate a clearly defined research agenda for the mucosal immunology of ASD. The primary translational priority is confirmation of the IgA1–IL-1β correlational association in an independent, adequately powered cohort. Based on the effect sizes observed here (full-cohort ρ  =  0.409), a future study targeting 90% power at α  =  0.01 (single pre-specified pair, one-tailed) would require *n*  ≥  70 complete pairs with both IgA1 and IL-1β/TP measured. This is achievable within a multi-centre European study enrolling 150–200 children. Such a study should additionally incorporate: (1) standardised faecal cytokine handling protocols with documented freeze–thaw stability data; (2) sex-stratified analyses or sex-balanced recruitment; (3) dietary assessment using validated food frequency questionnaires or food records; (4) concurrent gut microbiome characterisation (16S rRNA or shotgun metagenomics) to enable three-way partial correlation modelling; (5) longitudinal sampling to assess stability of the IgA1–IL-1β relationship over time in individual children.

A related open question, noted here with appropriate caution, is why the IgA1–IL-1β association was observed for the IgA1 subclass rather than IgA2, despite IgA2 predominating in the distal gut. This may reflect subclass differences in NF-κB-dependent class-switch regulation, or simply differential assay sensitivity; it remains speculative and requires dedicated investigation in a larger, prospectively designed cohort [8,29].

A secondary priority is understanding the mechanistic basis of the calprotectin subunit specificity observed here: why does S100A8 monomer (CAL-1) show a nominally significant association with IL-1β while the S100A8/S100A9 heterodimer (CAL1+2) and S100A9 monomer (CAL-2) do not? This unexpected finding may reflect differences in the in vivo stoichiometry of S100A8 and S100A9 release from neutrophils under different inflammatory conditions [30], or may reflect differential ex vivo stability of the monomers versus the heterodimer in the fecal matrix. Resolution of this question has implications for the use of calprotectin as a biomarker in ASD-related gut inflammation research and for the interpretation of commercial calprotectin assays, which typically target different epitopes.

A tertiary priority is the investigation of the inverse CARS–IL-1β/TP trend. This exploratory finding—the most clinically suggestive result in the dataset—should be prospectively tested in a study specifically powered for this endpoint (requiring *n * ≈  80 ASD children with complete CARS and IL-1β/TP data, based on ρ  =  −0.336 and 80% power at α  =  0.05 for a single pair). Such a study should concurrently measure IL-1Ra, IL-10, and other immunosuppressive mediators to test the compensatory hypothesis, and should assess dietary patterns and gut microbiome composition as potential confounders of the severity–inflammation relationship.

Beyond biomarker discovery, mucosal immune findings of the type reported here are relevant to the broader therapeutic landscape for ASD, which increasingly incorporates interventions targeting the gut–brain axis alongside established behavioural and rehabilitative approaches; pro-inflammatory mediators of the IL-1 and TNF families, and the chemokine CXCL8, have been proposed as candidate targets in this context [31]. Should the IgA1–IL-1β axis be confirmed in future work, it could inform the rational design or monitoring of such gut-directed adjunctive interventions.

## 4. Materials and Methods

### 4.1. Study Design and Setting

This was a cross-sectional, single-centre observational study conducted at the University Medical Centre Ljubljana (UMCL), Slovenia, with enrolment spanning 2016–2022. The study was approved by the Slovenian National Medical Ethics Committee (Protocol 0120-201/2016/6, approval granted 23 March 2016; Annex approved February 2022) and was supported by the Slovenian Research Agency (ARRS) grant J3-1756. All participants provided written informed consent (parents/guardians) or age-appropriate assent. The study adhered to the principles of the Declaration of Helsinki and is reported in accordance with the STROBE checklist (Supplementary Table S4).

### 4.2. Participants

Children aged 5–18 years were recruited from two groups. The ASD group (*n* =  74) comprised children with a diagnosis of ASD established according to DSM-5 criteria by a certified child psychiatrist at the Centre for Autism, University Children’s Hospital, UMCL. The diagnostic process included standardised assessment using the Autism Diagnostic Observation Schedule (ADOS-2) or Autism Diagnostic Interview-Revised (ADI-R) in addition to the DSM-5 clinical criteria. The control group (*n*  =  41) comprised neurotypical children without developmental, psychiatric, or chronic inflammatory conditions, recruited through paediatric outpatient clinics and local schools.

Exclusion criteria for both groups included: current antibiotic use within the preceding four weeks, use of systemic corticosteroids or other immunomodulatory agents, active inflammatory bowel disease or celiac disease, and acute infectious illness at time of sample collection. Probiotics or prebiotics taken within the preceding two weeks were also considered exclusion criteria given potential effects on stool immune marker concentrations. Written informed consent was obtained from parents or legal guardians prior to study entry. Participant flow, including reasons for exclusion, is summarised in Supplementary Figure S1 (STROBE item 13).

### 4.3. Clinical Assessment (CARS)

ASD severity was assessed using the Childhood Autism Rating Scale (CARS), a validated 15-item clinician-administered instrument in which each item is rated on a 4-point scale (1–4), yielding total scores ranging from 15 to 60 [32]. CARS scores were categorised as: below 30, consistent with non-autistic range (*n*  =  22); 30–36.5, mild to moderate ASD; and ≥37, severe ASD, per standard cut-points. CARS was available for all 74 ASD children (median 35.2, IQR 29.5–40.0). CARS was additionally recorded for all 41 control children to confirm neurotypical status; as expected, all controls scored below 30 (median 15.0, IQR 15.0–17.5; range 15.0–28.0), consistent with the non-autistic range.

### 4.4. Stool Collection and Processing

Fresh stool samples were collected by parents/guardians into sterile containers and transported to the laboratory within two hours of defecation. Stool homogenates were prepared at a 1:3 weight-to-volume ratio in phosphate-buffered saline (PBS, pH 7.4) and centrifuged (10,000× *g*, 10 min, 4 °C) to obtain a clarified supernatant. Aliquots were stored at −80 °C until analysis. Collection occurred between 2016 and 2022; all samples within a study year were processed in a single freeze–thaw cycle, and no more than two freeze–thaw cycles were applied to any sample used in the final analysis. Total protein concentration was measured by two independent methods: by bicinchoninic acid (BCA) proteinassay (Thermo Fisher Scientific, Waltham, MA, USA) for normalisation of proteomics data, and by Vitros Total Protein assay on the VITROS Chemistry System (Ortho Clinical Diagnostics, Raritan, NJ, USA) for normalisation of cytokine data. Vitros measurements were unavailable for 25 samples (21.7% of total), which are excluded from all cytokine analyses.

The decision to use two different total protein methods for normalisation (BCA for proteomics, Vitros for cytokines) reflects the practical reality that the proteomics and cytokine assays were performed in different laboratories at different time points. Both methods were used in their respective analytical pipelines from the outset. The correlation between BCA and Vitros total protein values in the subset of samples with both measurements (*n*  =  90) was Spearman ρ  =  0.87 (*p*  <  0.001), confirming that both methods capture the same underlying total protein concentration with high concordance. The use of different normalisation denominators for proteins and cytokines has the theoretical implication that protein–cytokine correlations incorporate assay-specific measurement error in the denominator; however, this measurement error is expected to be non-differential (i.e., unrelated to the specific protein or cytokine being measured), and would bias correlations toward zero rather than artifactually inflate them.

### 4.5. UHPLC-MS/MS Targeted Proteomics (RECETOX)

Stool immune proteins were quantified by ultra-high-performance liquid chromatography–tandem mass spectrometry (UHPLC-MS/MS) using a targeted dSRM (differential selected reaction monitoring) method on an Agilent 1290 Infinity II UHPLC system coupled to an Agilent Jet Stream (AJS) 6495A triple-quadrupole mass spectrometer (Agilent Technologies, Santa Clara, CA, USA) at RECETOX, Masaryk University, Brno, Czech Republic. Stable isotope-labelled (SIL) internal standards were used for all target proteins (listed in Supplementary Table S1). The SRM transitions are listed in Supplementary Table S2. The analyte panel comprised: IgA1, IgA2, IgA1+2 (total IgA), A1AT-1 (alpha-1-antitrypsin), CAL-1 (S100A8 monomer), CAL-2 (S100A9), and CAL1+2 (S100A8/S100A9 heterodimer). Results are expressed as ng/mg BCA-measured total protein.

For IgA subclass quantification, unique signature peptides within the IgA1 and IgA2 heavy chain constant regions were selected to ensure subclass specificity. The IgA1+2 signal was computed as the arithmetic sum of the IgA1 and IgA2 signals after individual quantification; it was not measured by a separate multiplex method. Peptide selection, analytical validation (linearity, recovery, precision, matrix effects), and quantification procedures are described in detail in the companion proteomics paper [20]; the selected peptides and their SRM transitions are listed in Supplementary Tables S1 and S2.

### 4.6. Luminex Multiplex Cytokine Assay

Stool cytokines were measured using the Bio-Plex Pro™ Human Cytokine 10-plex Assay (Bio-Rad Laboratories, Hercules, CA, USA) on a Bio-Plex® 200 System (Bio-Rad Laboratories, Hercules, CA, USA). The panel comprised: Eotaxin (CCL11), IFN-γ, IL-1α, IL-1β, IL-4, IL-6, IL-8, IL-10, IL-17, and TNF. A further cytokine, IL-15, was above the upper limit of quantification or missing in ≥74% of samples and was excluded from analysis. All cytokine concentrations were normalised to Vitros total protein and are expressed as pg/mg total protein (pg/mg TP). Measurements were performed in duplicate; coefficients of variation exceeding 20% were flagged and excluded.

The Luminex assay was performed on thawed stool homogenate supernatants. Each sample was run at two dilutions (1:1 and 1:3 in assay buffer) to enable back-calculation for samples with high cytokine concentrations. Assay performance was monitored using the manufacturer-supplied kit controls at all three concentration levels (low, medium, high). Results reported by the instrument as below the limit of detection were excluded from analysis rather than substituted. For IL-1β this affected 19.1% of the analysed cohort (Supplementary Table S3), and it is the principal reason why the effective sample size for pairs involving IL-1β is appreciably smaller than the cohort size. The intra-assay CV was <15% for all analytes and all control concentration levels.

### 4.7. Statistical Methods

All statistical analyses were performed in R version 4.3.1 [33].

Normality testing. Shapiro–Wilk tests were applied to all 17 normalised analytes in the full cohort, ASD subgroup, and control subgroup. The results confirmed severe non-normality for 16 of 17 markers (*p*  <  0.0002), justifying the exclusive use of non-parametric methods.

Group comparisons. Continuous variables (age) were compared between groups using the Mann–Whitney U test. Categorical variables (sex) were compared using the Pearson chi-square test.

Partial Spearman correlations. Age-adjusted partial Spearman rank correlations were computed using the Conover–Iman rank-transform method [28], in which Spearman correlations are approximated by computing Pearson partial correlations on ranked data after residualising the ranks of each variable against the rank of the covariate (age). A custom R function was validated against results from the ppcor package. For the sensitivity analysis, both age and sex (coded as a binary variable) were included as covariates.

Multiple testing correction. Benjamini–Hochberg FDR correction [34] was applied within each analytical stratum (full cohort, ASD, controls, CARS) across all 70 protein–cytokine pairs per stratum (7 proteins × 10 cytokines).

Bootstrap confidence intervals. Bootstrap 95% confidence intervals for partial Spearman correlation coefficients were computed using 2000 resamples with replacement and the percentile method.

Fisher r-to-z test. Between-group differences in correlation coefficients were evaluated using the Fisher r-to-z transformation, yielding a two-tailed z-statistic and corresponding *p*-value.

Missing data. Values reported below the assay limit of detection were treated as missing rather than substituted (Section 4.6), and all missing values were handled by pairwise deletion. The effective sample size for each protein–cytokine pair is reported in Table 3, Table 4 and Table 5. Missing data patterns were examined to assess whether missingness was related to group (ASD vs. control) or to other observed covariates, as a precaution against informative missing data bias.

Visualisation. Plots were produced using the ggplot2 package (version 3.4) [33]. Scatter plots with partial regression lines (after residualising both protein and cytokine on age) were produced for each pair in Table 3, stratified by group. Heatmaps of partial correlation matrices were constructed for each analytical stratum using the corrplot package.

Software and reproducibility. The complete analysis code and intermediate data products are available from the corresponding author upon request. The analysis was conducted in R 4.3.1 running under Ubuntu 22.04. The random seed for bootstrap resampling was set to 42 for reproducibility. All *p*-values are two-tailed unless otherwise specified.

## 5. Conclusions

This study demonstrates a consistently positive partial correlation between stool IgA1 and IL-1β/TP in a cohort of 115 children aged 5–18 years, observed across the full cohort and both diagnostic subgroups after age adjustment. No protein–cytokine pair survived Benjamini–Hochberg FDR correction in any stratum, and no formally significant between-group difference in correlation magnitude was confirmed (Fisher r-to-z: z  =  1.70, *p*  =  0.090). These findings should therefore be considered hypothesis-generating rather than confirmatory. IL-1β emerges as a candidate correlational hub in the gut mucosal immune network that warrants further investigation in adequately powered, prospective, multi-centre studies incorporating cytokine validation, sex-stratified analyses, dietary assessment, and gut microbiome characterisation.

The present study makes a methodological contribution by demonstrating the feasibility and statistical framework for within-cohort cross-assay partial correlation analysis in paediatric gut immunology, and by providing bootstrap-calibrated effect size estimates that can inform the sample size planning of future confirmatory studies. The honest reporting of null FDR results, combined with transparent presentation of exploratory trends and their confidence intervals, sets a standard for biomarker reporting in this field that prioritises scientific integrity over narrative simplicity. The IgA1–IL-1β/TP exploratory mucosal axis, observed in both ASD and control children with no confirmed between-group difference at current sample sizes, represents the most promising lead for further investigation and provides the primary hypothesis for a future confirmatory study. The CARS–IL-1β nominal trend, while intriguing, must be regarded as purely exploratory until replicated in an independently powered sample with pre-specified CARS analysis as a primary endpoint.

## Figures and Tables

**Table 1:** Demographic and clinical characteristics of the study cohort.

Characteristic	ASD (*n* = 74)	Controls (*n* = 41)	*p*-Value
Age, years, median [IQR]	9.8 [6.7–12.8]	10.8 [7.8–12.6]	0.317
Sex, male/female	59/15 (79.7% M)	21/20 (51.2% M)	0.003
CARS score, median [IQR]	35.2 [29.5–40.0]	15.0 [15.0–17.5]	—
CARS < 30	22 (29.7%)	41 (100%)	—
CARS ≥ 30	52 (70.3%)	0	—

(1) Age is given as median [interquartile range] and compared between groups by Mann–Whitney U test. (2) Sex is given as male/female counts with the percentage male, compared by Pearson chi-square test. (3) CARS = Childhood Autism Rating Scale, a 15-item clinician-administered instrument scored 15–60 (Section 4.3); it was administered to all 74 ASD and all 41 control children. (4) The conventional CARS cut-point of 30 separates the non-autistic from the autistic range; 22 ASD children scored below 30 and were nevertheless retained in the ASD group because each carried a DSM-5 diagnosis made by a certified child psychiatrist, a criterion that does not always align with older CARS scoring conventions (Section 2.7). (5) No *p*-value is reported for CARS because the instrument contributed to group assignment, so a between-group test on it would be circular. IQR: interquartile range.

## Data Availability

The datasets used and/or analysed during the current study are available from the corresponding author upon reasonable request. Anonymised individual-level data for the 115 participants, including stool proteomics values, cytokine concentrations, CARS scores, and demographic variables, can be shared with qualified researchers who provide a methodologically sound analysis plan and an institutional ethics approval for secondary use.

## Supplementary Materials

The following supporting information can be downloaded at: https://www.mdpi.com/article/10.3390/ijms27167097/s1.
