# Supplementary material for: Stool Mucosal Immune Protein–Cytokine Interconnections in Children with Autism Spectrum Disorder: An Age-Adjusted Partial Correlation Analysis
Source: Int J Mol Sci. 2026 Aug 7;27(16):7097. doi: 10.3390/ijms27167097 (PMC13513909; doi:10.3390/ijms27167097)
Supplement: Supplementary file 1 [file ijms-27-07097-s001.zip › ijms-4444033-supplementary.pdf]

Supplementary Materials

Stool Mucosal Immune Protein–Cytokine Interconnections in Children with Autism Spectrum Disorder

Table S1. Stable isotope-labelled (SIL) internal standard peptides used for SRM-based quantification.

Protein	Peptide sequence	Isotope label	Spike conc.
IgA1	ASGYTFTSY[+10]WMHWVR	13C6 15N4-Arg	5 fmol/µL
IgA2	VSSASTK[+8]GPSIFR	13C6 15N2-Lys	5 fmol/µL
CAL-1 (S100A8)	LLETECPQYIR[+10]	13C6 15N4-Arg	10 fmol/µL
CAL-2 (S100A9)	NIETIINTFHQYSVK[+8]	13C6 15N2-Lys	10 fmol/µL
A1AT	EIGELYLPK[+8]	13C6 15N2-Lys	5 fmol/µL
Lcn-2	VFSNSDTHLLSLPQPEFR[+10]	13C6 15N4-Arg	5 fmol/µL
HP	TEGDGVYTLNNEK[+8]	13C6 15N2-Lys	5 fmol/µL

SIL, stable isotope-labelled; SRM, selected reaction monitoring; Arg, arginine; Lys, lysine.

Table S2. SRM transitions for quantification of stool immune proteins on the Agilent 6495A triple-quadrupole mass spectrometer.

Protein	Peptide	Q1 (Da)	Q3 (Da)	CE (V)	Type
IgA1	ASGYTFTSY[+10]WMHWVR	816.4	1015.5	28	Quantifier
IgA1	ASGYTFTSY[+10]WMHWVR	816.4	952.5	28	Qualifier
IgA2	VSSASTK[+8]GPSIFR	778.9	1021.5	24	Quantifier
IgA2	VSSASTK[+8]GPSIFR	778.9	870.5	24	Qualifier
CAL-1 (S100A8)	LLETECPQYIR[+10]	702.4	984.5	26	Quantifier
CAL-1 (S100A8)	LLETECPQYIR[+10]	702.4	856.4	26	Qualifier
CAL-2 (S100A9)	NIETIINTFHQYSVK[+8]	604.3	945.5	22	Quantifier
CAL-2 (S100A9)	NIETIINTFHQYSVK[+8]	604.3	832.4	22	Qualifier
A1AT	EIGELYLPK[+8]	540.3	794.5	20	Quantifier
A1AT	EIGELYLPK[+8]	540.3	681.4	20	Qualifier
Lcn-2	VFSNSDTHLLSLPQPEFR[+10]	1057.6	1321.7	38	Quantifier
Lcn-2	VFSNSDTHLLSLPQPEFR[+10]	1057.6	1208.6	38	Qualifier
HP	TEGDGVYTLNNEK[+8]	757.4	1003.5	26	Quantifier
HP	TEGDGVYTLNNEK[+8]	757.4	890.4	26	Qualifier

Q1, precursor ion; Q3, product ion; CE, collision energy; A1AT, alpha-1-antitrypsin; CAL, calprotectin subunit; HP, haptoglobin; Lcn-2, lipocalin-2. Haptoglobin and lipocalin-2 form part of the targeted SRM method but are not among the analytes reported in this study; the analysed panel is the seven proteins listed in Section 4.5.

Table S3. Percentage of stool samples above the lower limit of detection (LOD) and above the lower limit of quantification (LOQ), by cytokine/chemokine and diagnostic group.

Analyte	Group	n assayed	Below LOD (n)	Above LOD, below LOQ (n)	Above LOQ (n)	Above LOD (%)	Above LOQ (%)
Eotaxin/CCL11	Full cohort	115	2	18	95	98.3	82.6
Eotaxin/CCL11	ASD	74	2	14	58	97.3	78.4
Eotaxin/CCL11	Control	41	0	4	37	100.0	90.2
IFN-γ	Full cohort	115	22	35	58	80.9	50.4
IFN-γ	ASD	74	17	22	35	77.0	47.3
IFN-γ	Control	41	5	13	23	87.8	56.1
IL-1α	Full cohort	115	1	2	112	99.1	97.4
IL-1α	ASD	74	1	2	71	98.6	95.9
IL-1α	Control	41	0	0	41	100.0	100.0
IL-1β	Full cohort	115	22	70	23	80.9	20.0
IL-1β	ASD	74	18	43	13	75.7	17.6
IL-1β	Control	41	4	27	10	90.2	24.4
IL-4	Full cohort	115	47	18	50	59.1	43.5
IL-4	ASD	74	27	15	32	63.5	43.2
IL-4	Control	41	20	3	18	51.2	43.9
IL-6	Full cohort	115	17	23	75	85.2	65.2
IL-6	ASD	74	13	11	50	82.4	67.6
IL-6	Control	41	4	12	25	90.2	61.0
IL-8/CXCL8	Full cohort	115	20	74	21	82.6	18.3
IL-8/CXCL8	ASD	74	15	50	9	79.7	12.2
IL-8/CXCL8	Control	41	5	24	12	87.8	29.3
IL-10	Full cohort	115	50	38	27	56.5	23.5
IL-10	ASD	74	29	25	20	60.8	27.0
IL-10	Control	41	21	13	7	48.8	17.1
IL-17	Full cohort	115	2	88	25	98.3	21.7
IL-17	ASD	74	1	54	19	98.6	25.7
IL-17	Control	41	1	34	6	97.6	14.6
TNF	Full cohort	115	22	36	57	80.9	49.6
TNF	ASD	74	15	22	37	79.7	50.0
TNF	Control	41	7	14	20	82.9	48.8

Legend. n assayed = number of children in that group with a recorded Luminex measurement for that analyte. Below LOD (n) = results reported by the instrument as out of range below the assay's lower limit of detection ("OOR <"); these were excluded from analysis rather than substituted, which is the principal reason the effective sample size for pairs involving IL-1β is well below the cohort size. Above LOD, below LOQ (n) = results with a detectable signal above the LOD but below the lower limit of quantification, for which concentrations were extrapolated below the validated range of the standard curve. Above LOQ (n, %) = results falling within the validated quantification range. Above LOD (%) combines the last two categories. Counts are given for the analysed cohort of 115 children (ASD n = 74, controls n = 41) as defined in Table 1, and the three status categories sum to n assayed in every row. Percentages are rounded to one decimal place.

Table S4. STROBE checklist for reporting of observational studies (cross-sectional studies).

Manuscript: "Stool Mucosal Immune Protein–Cytokine Interconnections in Children with Autism Spectrum Disorder: An Age-Adjusted Partial Correlation Analysis"

Authors: Osredkar J, Godnov U, Jekovec Vrhovšek M, Osredkar D, Avguštin G, Fabjan T, Kumer K

Target journal: International Journal of Molecular Sciences (IJMS, MDPI). Manuscript ijms-4444033.

Study design: Cross-sectional, single-centre observational cohort study (STROBE cross-sectional checklist version 4)

Item No.	Section / Topic	Recommendation (STROBE)	Location in Manuscript
**1**	Title & Abstract	(a) Indicate the study's design with a commonly used term in the title or the abstract<br>(b) Provide in the abstract an informative and balanced summary of what was done and what was found	Title: "…An Age-Adjusted Partial Correlation Analysis" indicates the cross-sectional, observational design. Abstract: structured as Background, Methods, Results and Conclusions. States the cohort (115 children; 74 ASD, 41 controls, aged 5–18 years), the 70-pair correction family, the principal null result, and the exploratory IgA1–IL-1β/TP association with its confidence interval.
**2**	Introduction Background/rationale	Explain the scientific background and rationale for the investigation being reported	Section 1 (Introduction): epidemiology of ASD, gastrointestinal comorbidity, stool IgA subclasses, calprotectin subunits, α_1-antitrypsin and cytokine biology. Gap statement: whether specific cytokines and mucosal immune proteins are related within the same cohort has not been formally examined.
**3**	Introduction Objectives	State specific objectives, including any prespecified hypotheses	Section 1, final paragraph: three aims — (1) age-adjusted partial Spearman correlations across all protein–cytokine pairs in the full cohort and in both diagnostic subgroups; (2) identification of any axis showing group specificity; (3) association of stool markers with CARS severity within the ASD subgroup.<br>No hypothesis was pre-registered and no analysis was pre-specified in a public protocol. The analytic hierarchy is described as exploratory throughout (Sections 2.5, 2.6 and 3.1), and the between-group comparison is identified as a single exploratory test (Section 3.7, item 8).
**4**	Methods Study design	Present key elements of study design early in the paper	Section 4.1: "This was a cross-sectional, single-centre observational study conducted at the University Medical Centre Ljubljana (UMCL), Slovenia."
**5**	Methods Setting	Describe the setting, locations, and relevant dates, including periods of recruitment, exposure, follow-up, and data collection	Section 4.1: University Medical Centre Ljubljana, Slovenia; enrolment spanning 2016–2022. Ethics approval by the Slovenian National Medical Ethics Committee, Protocol 0120-201/2016/6, granted 23 March 2016, with an Annex approved February 2022 (also in the Institutional Review Board Statement). Stool collection and processing dates are given in Section 4.4. There is no follow-up: each child contributed a single sample.
**6**	Methods Participants	Cross-sectional study — give the eligibility criteria, and the sources and methods of participant selection	Section 4.2: the ASD group (n = 74) comprised children aged 5–18 years with a DSM-5 diagnosis established by a certified child psychiatrist at the Centre for Autism, University Children's Hospital, UMCL, supported by

Item No.	Section / Topic	Recommendation (STROBE)	Location in Manuscript
			ADOS-2 or ADI-R. The control group (n = 41) comprised neurotypical children without developmental, psychiatric or chronic inflammatory conditions, recruited through paediatric outpatient clinics and local schools.<br>Exclusion criteria for both groups: antibiotic use within the preceding four weeks; systemic corticosteroids or other immunomodulatory agents; active inflammatory bowel disease or coeliac disease; acute infectious illness at sampling; and probiotics or prebiotics within the preceding two weeks. The analysed cohort is the set of children with both proteomics and cytokine data; the flow is given in Figure S1.
**7**	Methods Variables	Clearly define all outcomes, exposures, predictors, potential confounders, and effect modifiers. Give diagnostic criteria, if applicable	Sections 4.5 and 4.6, with Tables S1 and S2. Seven stool immune proteins by UHPLC-MS/MS: IgA1, IgA2, IgA1+2 (the arithmetic sum), A1AT-1, CAL-1 (S100A8 monomer), CAL-2 (S100A9) and CAL1+2 (the heterodimer), expressed as ng/mg BCA total protein. Ten cytokines and chemokines by Luminex: Eotaxin/CCL11, IFN-γ, IL-1α, IL-1β, IL-4, IL-6, IL-8/CXCL8, IL-10, IL-17 and TNF, expressed as pg/mg Vitros total protein. IL-15 was excluded before analysis, being above the upper limit of quantification or missing in ≥74% of samples.<br>Age is a continuous covariate in every partial correlation. Sex is added as a second covariate in the sensitivity analysis of Section 2.5. CARS total score is the severity variable of Section 2.7 and is defined in Section 4.3.
**8**	Methods Data sources / measurement	For each variable of interest give sources of data and details of methods of assessment (measurement). Describe comparability of assessment methods if there is more than one group	Sections 4.4 to 4.6. Stool collected into sterile containers and transported within two hours; homogenates prepared at 1:3 (w/v) in PBS pH 7.4, centrifuged at 10,000 × g for 10 min at 4 °C, aliquots stored at −80 °C, with no more than two freeze–thaw cycles for any analysed sample.<br>Proteins quantified by targeted dSRM UHPLC-MS/MS on an Agilent AJS 6495A triple-quadrupole instrument at RECETOX, Masaryk University, with stable isotope-labelled internal standards (Tables S1 and S2).<br>Cytokines measured with the Bio-Plex Pro Human Cytokine 10-plex assay on a Bio-Plex 200 system, in duplicate, at two dilutions.<br>Both assays were applied identically to the two diagnostic groups. Total protein was measured by two methods for two purposes — BCA for the proteomics and Vitros for the cytokines — because the assays were run in different laboratories; their concordance is reported in Section 4.4 (Spearman ρ = 0.87, n = 90).
**9**	Methods Bias	Describe any efforts taken to address potential sources of bias	Section 4.7. Age is carried as a continuous covariate in every partial correlation, so developmental confounding is removed by design rather than by matching. Cytokines are normalised to total protein to limit stool-consistency bias. Results reported below the assay limit of detection were excluded rather than substituted at LOD/2 (Sections

Item No.	Section / Topic	Recommendation (STROBE)	Location in Manuscript
			4.6 and 4.7; this description was corrected at revision). Children without a Vitros total protein result (21.7% of samples) could not enter the cytokine analyses. Two further sources of bias are addressed analytically rather than by design: the sex imbalance between groups, by the sex-adjusted sensitivity analysis of Section 2.5, and the pairwise deletion of missing cytokine values, by the multiple-imputation analysis of Section 2.6.
**10**	Methods Study size	Explain how the study size was arrived at	Sections 4.1 and 4.2: the cohort is the set of children with both proteomics and cytokine measurements at the same time point, giving n = 115. No a priori power calculation was performed. The consequences are discussed in Section 3.4 and listed in Section 3.7 as items 7 (underpowered subgroup analyses) and 9 (no replication cohort).
**11**	Methods Quantitative variables	Explain how quantitative variables were handled in the analyses. If applicable, describe which groupings were chosen and why	Sections 4.5 to 4.7. All analytes are treated as continuous. Age is retained as a continuous covariate with no dichotomisation, and CARS as a continuous severity score; the CARS cut-points of Section 4.3 are used to describe the cohort, not to group it for analysis. Benjamini–Hochberg FDR correction was applied across the 70 protein–cytokine pairs (7 proteins × 10 cytokines) within each stratum independently, and separately across the family of 17 marker tests for the CARS analysis of Table 5.
**12**	Methods Statistical methods	(a) Describe all statistical methods, including those used to control for confounding<br>(b) Describe any methods used to examine subgroups and interactions<br>(c) Explain how missing data were addressed<br>(d) Cross-sectional study — if applicable, describe analytical methods taking account of sampling strategy<br>(e) Describe any sensitivity analyses	(a) Age-adjusted partial Spearman correlation by the Conover–Iman rank transform (Conover & Iman 1981), with Benjamini–Hochberg FDR control (Benjamini & Hochberg 1995); Mann–Whitney U and χ^2 for the group comparisons of Table 1; bootstrap 95% confidence intervals, percentile method, 2000 resamples; Fisher r-to-z for the single between-group comparison. R 4.3.1, ggplot2 3.4, random seed 42 (Section 4.7).<br>(b) Three strata are analysed separately — full cohort, ASD and controls (Sections 2.3 and 2.4) — each forming its own correction family, plus the CARS analysis within the ASD subgroup (Section 2.7). No interaction terms were fitted; the ASD-versus-control comparison is a single exploratory Fisher r-to-z test ($z = 1.70$, $p = 0.090$).<br>(c) Pairwise deletion: each correlation uses the children with complete data for both variables, and the effective n is reported for every pair (n = 36–89 in the full cohort). The MCAR assumption this requires, and the evidence against it, are set out in Section 3.7, item 3.<br>(d) Single-centre consecutive clinical cohort; no sampling weights or design effects apply.<br>(e) Three sensitivity analyses: sex as an additional covariate (Section 2.5); multiple imputation by chained equations for the two central associations (Section 2.6; m = 30, predictive mean matching, 20 iterations); and

Item No.	Section / Topic	Recommendation (STROBE)	Location in Manuscript
			exclusion of the 22 ASD children scoring below the CARS cut-point (Section 2.7).
**13**	Results Participants	(a) Report numbers of individuals at each stage of study — e.g. numbers potentially eligible, examined, confirmed eligible, included in the study, completing follow-up, and analysed<br>(b) Give reasons for non-participation at each stage<br>(c) Consider use of a flow diagram	(a) Table 1: n = 115 analysed (ASD 74, controls 41). Section 4.2 describes how the cohort was derived from the larger proteomics and cytokine series.<br>(b) Exclusions and their reasons are listed in Section 4.2. The 22 ASD children scoring below the CARS cut-point were retained in the ASD group on clinical grounds, since diagnosis rested on DSM-5 criteria rather than on CARS (Section 2.7).<br>(c) Figure S1 is the STROBE participant flow diagram, showing enrolment, exclusions and analytical inclusion for each stratum. It was added at revision.
**14**	Results Descriptive data	(a) Give characteristics of study participants (e.g. demographic, clinical, social) and information on exposures and potential confounders<br>(b) Indicate number of participants with missing data for each variable of interest	(a) Table 1: median age 9.8 years [IQR 6.7–12.8] in ASD and 10.8 [7.8–12.6] in controls ($p = 0.317$, Mann–Whitney U); sex 59/15 male/female in ASD and 21/20 in controls ($\chi^2 = 8.82$, $p = 0.003$); CARS total median 35.2 [29.5–40.0] in ASD and 15.0 [15.0–17.5] in controls. The age row was corrected at revision.<br>(b) The effective n is given for every marker in Table 2 and for every pair in Tables 3 to 5. Missingness is highest for IL-10 (~44%) and IL-4 (~38%); Vitros total protein, required for cytokine normalisation, was unavailable for 21.7% of samples; IL-1β fell below the assay limit of detection in 19.1%. Table S3 gives the percentage above the LOD and above the LOQ for every analyte in every group.
**15**	Results Outcome data	Cross-sectional study — report summary measures and measures of association	Table 2 reports median [IQR] and n for all 17 markers by group. Tables 3 and 4 report the age-adjusted partial Spearman ρ, bootstrap 95% confidence interval, effective n, p-raw and p-FDR for the full cohort and for each subgroup; Table 5 does the same for the CARS analysis. Figure 1 is the complete 70-pair correlation matrix for the full cohort. Figures S2 to S4 give the subgroup matrices, the age-residualised scatter plots for the five strongest pairs, and the CARS correlations.
**16**	Results Main results	(a) Give unadjusted estimates and, if applicable, confounder-adjusted estimates and their precision (e.g., 95% CI). Make clear which confounders were adjusted for and why they were included<br>(b) Report category boundaries when continuous variables were categorised<br>(c) If relevant, consider translating estimates of relative risk into absolute risk for a meaningful time period	(a) Every coefficient reported is an age-adjusted partial Spearman ρ; age was chosen as the covariate because the cohort spans 5–18 years and both analyte classes vary with age. Unadjusted coefficients are not reported. Bootstrap 95% confidence intervals are given in Tables 3 and 4. No pair reached p-FDR < 0.05 in any stratum. The strongest association is IgA1–IL-1β/TP: $\rho = 0.409$ (95% CI 0.131–0.634, n = 62, p-FDR = 0.075, a trend) in the full cohort; $\rho = 0.583$ (95% CI 0.169–0.822, n = 30, p-FDR = 0.063) in controls; $\rho = 0.210$ (95% CI −0.190–0.577, n = 32, p-FDR = 0.961) in ASD, with no significant between-group difference (Fisher r-to-z: z = 1.70, p =

Item No.	Section / Topic	Recommendation (STROBE)	Location in Manuscript
			0.090). Multiple imputation attenuates the full-cohort estimate to ρ = 0.265 (95% CI 0.055–0.452).<br>(b) No continuous variable was categorised for analysis.<br>(c) Not applicable: the study reports correlation coefficients, not risk estimates.
**17**	Results Other analyses	Report other analyses done — e.g. analyses of subgroups and interactions, and sensitivity analyses	Subgroup analyses for ASD (n = 74) and controls (n = 41), each corrected within its own 70-pair family (Table 4; Figure S2). CARS severity analysis within the ASD subgroup, corrected across a separate family of 17 marker tests (Table 5; Figure S4).<br>Sensitivity analyses: sex as an additional covariate (Section 2.5); multiple imputation for the two central associations (Section 2.6); and exclusion of the 22 sub-cut-point CARS children (Section 2.7). No interaction tests were performed.
**18**	Discussion Key results	Summarise key results with reference to study objectives	Sections 3.1 to 3.6 and Section 5 (Conclusions). The principal result is null: no protein–cytokine pair survived Benjamini–Hochberg correction in any stratum. IL-1β is the cytokine most consistently associated with the immune proteins, and the IgA1–IL-1β/TP association is positive in all three strata; it is presented as an exploratory hypothesis, not a finding. The inverse CARS–IL-1β/TP correlation (ρ = −0.336, n = 41, p-raw = 0.034, p-FDR = 0.576) is nominal only.
**19**	Discussion Limitations	Discuss limitations of the study, taking into account sources of potential bias or imprecision. Discuss both direction and magnitude of any potential bias	Section 3.7 lists nine limitations: (1) cross-sectional design, so no causal inference; (2) single centre, tertiary referral setting; (3) high cytokine missingness with pairwise deletion; (4) unestablished validity of Luminex cytokine measurement in the stool matrix; (5) sex imbalance; (6) no dietary or supplement data; (7) underpowered subgroup analyses; (8) the between-group Fisher r-to-z test being exploratory rather than pre-specified; (9) no independent replication cohort.<br>Direction and magnitude of bias are stated where they can be judged. Item 3 reports that multiple imputation returns estimates about one third smaller than the complete-case values, with the CARS interval crossing zero, and concludes that the complete-case estimates are likely upward-biased and should be read as upper bounds. Item 4 reports, from Table S3, that several analytes have a high proportion of detectable values below the limit of quantification — IL-1β 80.9% above the LOD but only 20.0% above the LOQ, IL-8/CXCL8 82.6% and 18.3% — and that no spike-recovery or matrix-specific freeze–thaw stability data exist for this matrix, so differential recovery cannot be excluded as a partial contributor to the observed correlations.
**20**	Discussion Interpretation	Give a cautious overall interpretation of results considering objectives, limitations, multiplicity of analyses, and results from similar studies	Sections 3.1 to 3.6 and Section 5. Results are presented as hypothesis-generating throughout. Benjamini–

Item No.	Section / Topic	Recommendation (STROBE)	Location in Manuscript
			Hochberg correction was applied across the 70-pair family within each stratum and no pair survived it, a point made in the Abstract, in Section 2.3, in the legends to Tables 3 and 4, and in the Conclusions. The numerically weaker IgA1–IL-1β association in ASD is explicitly not interpreted as a group difference: the Fisher r-to-z test does not reach significance, and a difference in significance between two groups is not itself a significant difference (Section 3.2 and the legend to Table 4, ref [22]). The findings are placed against the published mucosal immune literature (refs [2, 3, 6–10, 12, 15, 17, 29]), and longitudinal multi-omic studies are set out as the next step in Section 3.9.
**21**	Discussion Generalisability	Discuss the generalisability (external validity) of the study results	Section 3.7, items 2 and 5, and Section 3.8. A single-centre Slovenian cohort recruited through a specialist autism centre carries a tertiary referral bias, and the sex distribution differs significantly between groups (79.7% male in ASD against 51.2% in controls). The results may not generalise to community-ascertained or ethnically more diverse ASD populations, and the subgroup sizes do not support sex-stratified analysis.
**22**	Other information Funding	Give the source of funding and the role of the funders for the present study and, if applicable, for the original study on which the present article is based	Funding statement (back matter) and Section 4.1: funded by the Slovenian Research Agency (ARRS), grant J3-1756. The funder had no role in study design, data collection and analysis, decision to publish, or preparation of the manuscript. The UHPLC-MS/MS proteomics analyses were performed at RECETOX, Masaryk University, Brno, a facility supported by the Czech Ministry of Education, Youth and Sports (LM2023069 RECETOX RI). The authors declare no conflicts of interest.

Note: This checklist was completed against the revised manuscript of 29 July 2026 and supersedes the version prepared for the draft of 8 June 2026. Section numbers refer to the revised manuscript, in which the Results are Section 2, the Discussion Section 3, the Materials and Methods Section 4, and the Conclusions Section 5. Item 13(c) is met by Figure S1, the STROBE participant flow diagram added at revision. STROBE reference: von Elm E, Altman DG, Egger M, Pocock SJ, Gøtzsche PC, Vandenbroucke JP; STROBE Initiative. The Strengthening the Reporting of Observational Studies in Epidemiology (STROBE) statement: guidelines for reporting observational studies. PLoS Med. 2007;4(10):e296. https://doi.org/10.1371/journal.pmed.0040

Table S5. Distributional statistics and Shapiro–Wilk normality tests for the 17 total-protein-normalised stool markers, by stratum.

Marker (unit)	Group	n	Skewness	Excess kurtosis	W	p
IgA1 (ng/mg)	Full cohort	93	5.38	36.10	0.469	<0.0001
IgA1 (ng/mg)	ASD	57	2.98	10.42	0.660	<0.0001
IgA1 (ng/mg)	Control	36	5.34	30.36	0.361	<0.0001
IgA2 (ng/mg)	Full cohort	108	6.86	59.93	0.449	<0.0001
IgA2 (ng/mg)	ASD	68	1.08	-0.03	0.842	<0.0001
IgA2 (ng/mg)	Control	40	5.54	33.08	0.367	<0.0001
IgA1+2 (ng/mg)	Full cohort	112	7.73	71.57	0.374	<0.0001
IgA1+2 (ng/mg)	ASD	72	1.65	2.13	0.787	<0.0001
IgA1+2 (ng/mg)	Control	40	5.86	35.94	0.309	<0.0001
A1AT-1 (ng/mg)	Full cohort	111	2.30	8.59	0.815	<0.0001
A1AT-1 (ng/mg)	ASD	71	2.33	7.52	0.794	<0.0001
A1AT-1 (ng/mg)	Control	40	0.48	-1.13	0.911	0.0041
CAL-1 (ng/mg)	Full cohort	109	4.37	22.69	0.469	<0.0001
CAL-1 (ng/mg)	ASD	70	3.86	16.82	0.499	<0.0001
CAL-1 (ng/mg)	Control	39	3.46	11.89	0.499	<0.0001
CAL-2 (ng/mg)	Full cohort	95	4.33	22.95	0.488	<0.0001
CAL-2 (ng/mg)	ASD	62	3.84	17.22	0.519	<0.0001
CAL-2 (ng/mg)	Control	33	3.20	10.03	0.524	<0.0001
CAL1+2 (ng/mg)	Full cohort	95	4.22	21.42	0.487	<0.0001
CAL1+2 (ng/mg)	ASD	62	3.74	16.04	0.518	<0.0001
CAL1+2 (ng/mg)	Control	33	3.21	9.96	0.510	<0.0001
Eotaxin/TP (pg/mg)	Full cohort	89	0.39	-0.28	0.972	0.0553
Eotaxin/TP (pg/mg)	ASD	52	0.38	-0.72	0.958	0.0653
Eotaxin/TP (pg/mg)	Control	37	0.47	0.85	0.967	0.3368
IFN-γ/TP (pg/mg)	Full cohort	74	4.44	24.78	0.556	<0.0001
IFN-γ/TP (pg/mg)	ASD	42	4.11	19.27	0.522	<0.0001
IFN-γ/TP (pg/mg)	Control	32	2.06	4.25	0.758	<0.0001

Marker (unit)	Group	n	Skewness	Excess kurtosis	W	p
IL-1α/TP (pg/mg)	Full cohort	90	3.07	12.11	0.695	<0.0001
IL-1α/TP (pg/mg)	ASD	53	2.80	8.72	0.670	<0.0001
IL-1α/TP (pg/mg)	Control	37	0.90	0.76	0.924	0.0149
IL-1β/TP (pg/mg)	Full cohort	74	6.64	48.46	0.260	<0.0001
IL-1β/TP (pg/mg)	ASD	41	5.48	32.57	0.361	<0.0001
IL-1β/TP (pg/mg)	Control	33	5.13	27.42	0.287	<0.0001
IL-4/TP (pg/mg)	Full cohort	55	3.67	15.94	0.595	<0.0001
IL-4/TP (pg/mg)	ASD	36	2.90	9.97	0.684	<0.0001
IL-4/TP (pg/mg)	Control	19	3.68	14.87	0.531	<0.0001
IL-6/TP (pg/mg)	Full cohort	77	1.22	2.62	0.923	0.0002
IL-6/TP (pg/mg)	ASD	44	0.68	0.12	0.961	0.1362
IL-6/TP (pg/mg)	Control	33	1.74	4.69	0.856	0.0005
IL-8/TP (pg/mg)	Full cohort	74	3.95	16.21	0.430	<0.0001
IL-8/TP (pg/mg)	ASD	42	4.17	18.22	0.393	<0.0001
IL-8/TP (pg/mg)	Control	32	3.87	16.70	0.479	<0.0001
IL-10/TP (pg/mg)	Full cohort	50	3.20	12.17	0.616	<0.0001
IL-10/TP (pg/mg)	ASD	32	2.19	4.44	0.701	<0.0001
IL-10/TP (pg/mg)	Control	18	3.52	13.54	0.528	<0.0001
IL-17/TP (pg/mg)	Full cohort	88	4.40	24.35	0.496	<0.0001
IL-17/TP (pg/mg)	ASD	52	2.68	7.38	0.627	<0.0001
IL-17/TP (pg/mg)	Control	36	4.54	22.77	0.409	<0.0001
TNF/TP (pg/mg)	Full cohort	71	3.32	11.20	0.436	<0.0001
TNF/TP (pg/mg)	ASD	40	3.18	10.08	0.452	<0.0001
TNF/TP (pg/mg)	Control	31	3.47	12.59	0.420	<0.0001

Legend. Shapiro–Wilk tests were applied to the untransformed, total-protein-normalised values of all 17 markers, separately in the full cohort and in each diagnostic group. n = number of children with a valid measurement for that marker in that stratum; values reported below the assay limit of detection were excluded rather than substituted (Section 4.6), so n varies by marker and the two group columns sum to the full-cohort column. Skewness and excess kurtosis are the Fisher–Pearson standardised moment coefficients, both zero under normality. W is the Shapiro–Wilk statistic and p the associated two-sided p-value, uncorrected for multiple testing. Sixteen of the 17 markers deviated significantly from normality in the full cohort (all $p \leq 0.0002$, and 15 of them at $p < 0.0001$); Eotaxin/TP was the sole

marker compatible with normality (W = 0.973, p = 0.055) and the only one with skewness below 1.0. These results are the basis for the exclusive use of non-parametric methods throughout (Sections 2.2 and 4.7). TP: total protein.

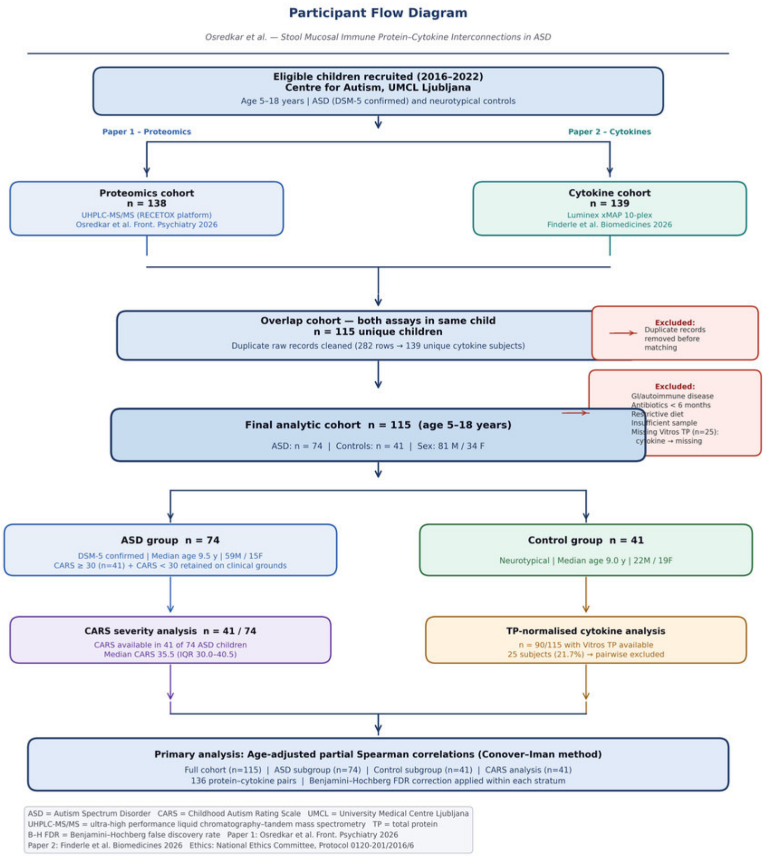

Figure S1. STROBE participant flow diagram showing enrolment, exclusions, and analytical inclusion for each analytical stratum.

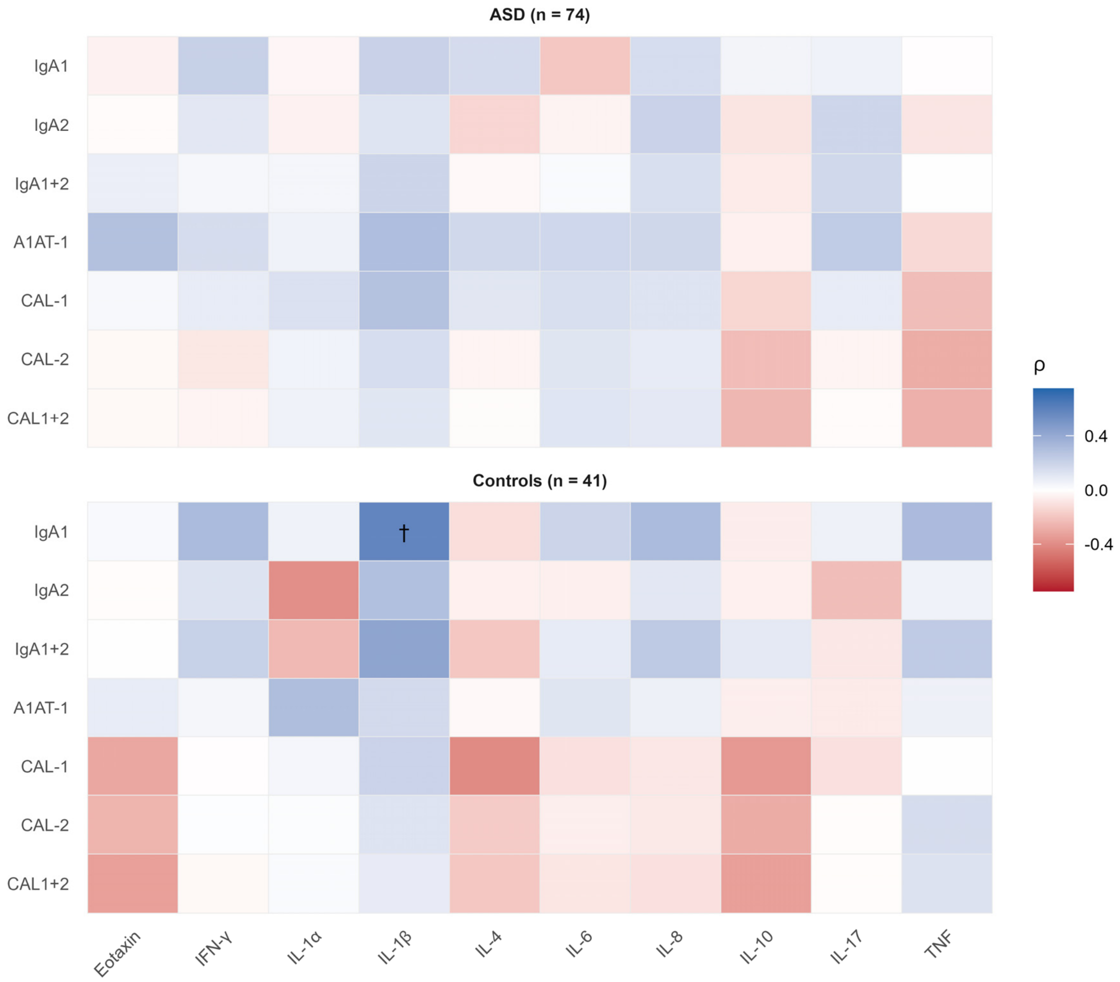

Figure S2. Age-adjusted partial Spearman correlations by diagnostic group (ASD n = 74; Controls n = 41).

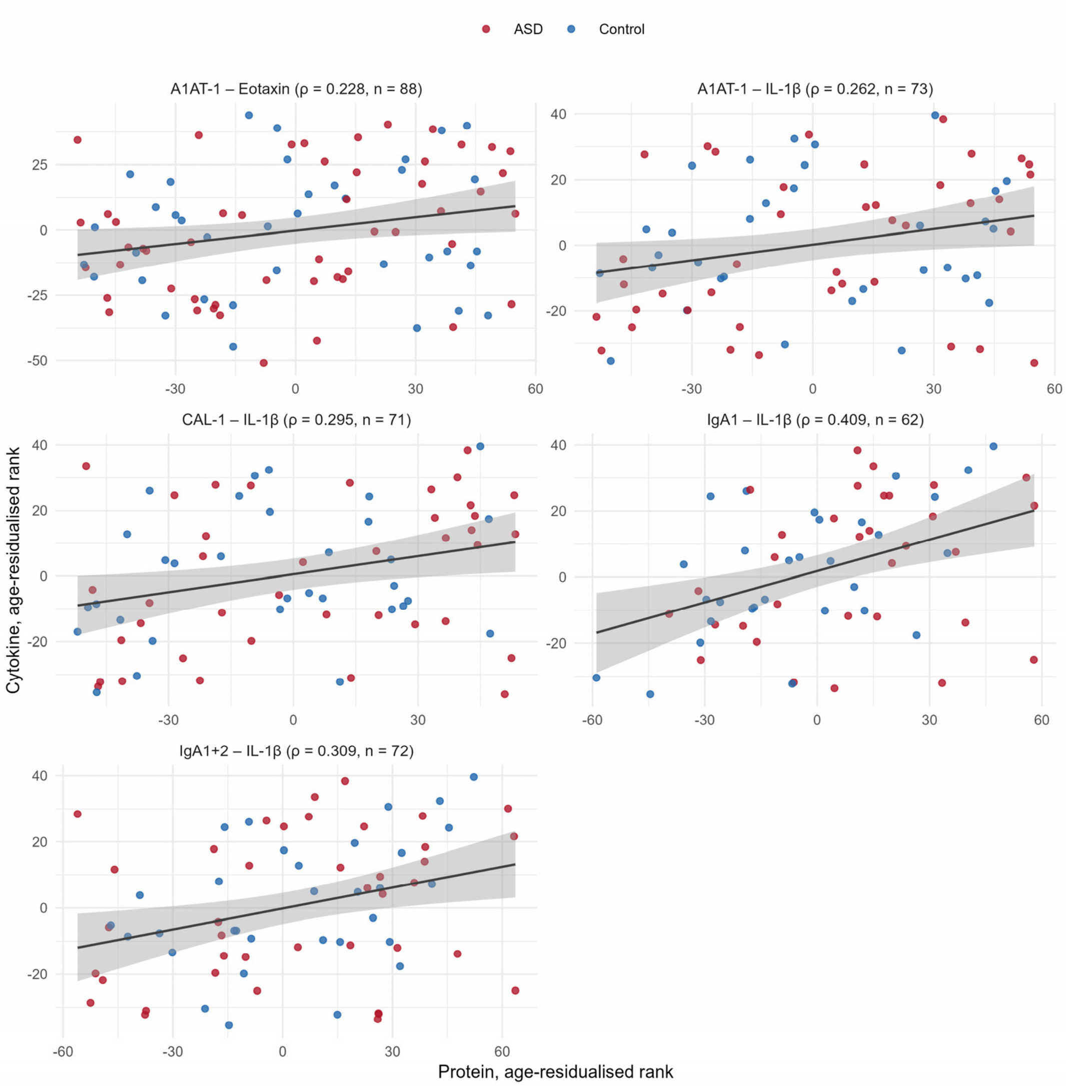

Figure S3. Age-residualised scatter plots for the five protein–cytokine pairs with the strongest associations in the full cohort.

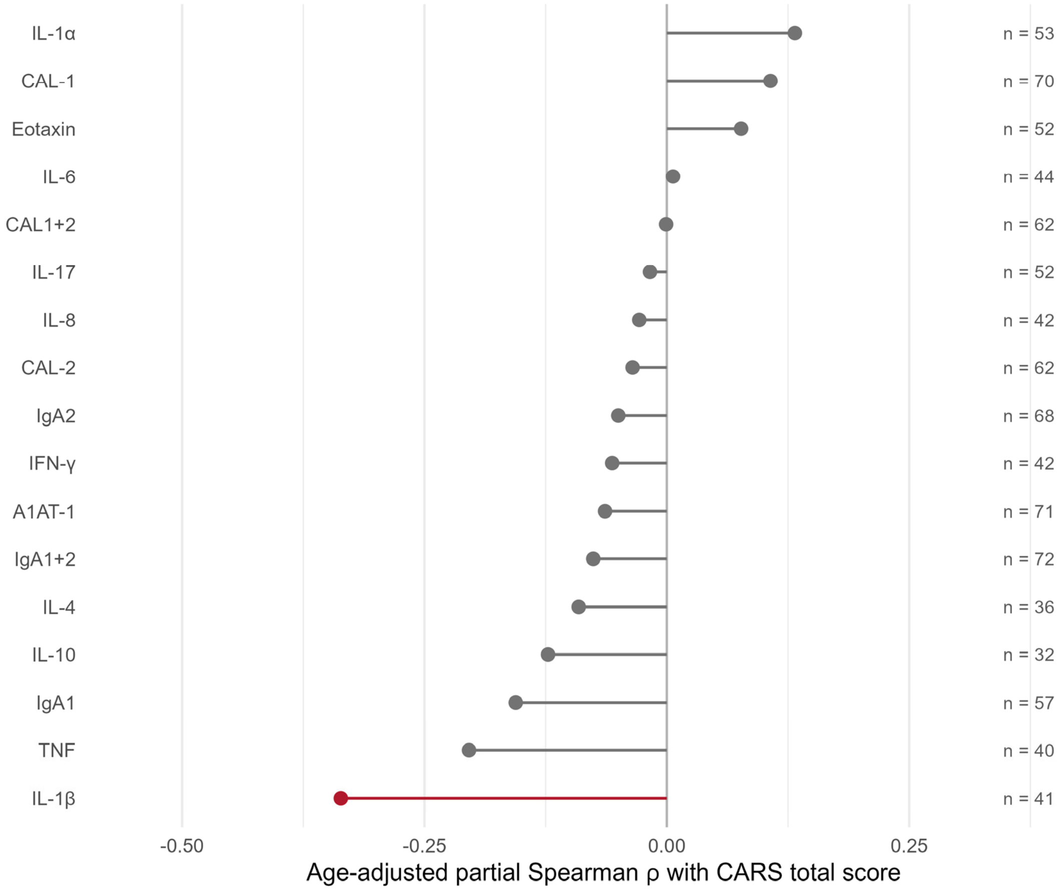

Figure S4. Age-adjusted partial Spearman correlations between stool markers and CARS total score (ASD subgroup).
